# Great Dental Patent Case

**Published:** 1855-07

**Authors:** J. Allen

**Affiliations:** 30 Bond Street, New York


### GREAT DENTAL PATENT CASE.

IN THE U. S. CIRCUIT COURT for the Southern District of Ohio.

CINCINNATI, O., May 11th—19th, 1855.

*Suit brought by Dr. John Allen against Dr. William M. Hunter, for an alleged Infringement of his Patent "for a new and useful improvement in setting Mineral Teeth."*

This case came on for trial Friday morning, May 11th, in the U. S. Circuit Court, for the Southern District of Ohio. Present, Hon. JOHN McLEAN, one of the Justices of the Supreme Court of the United States, and Hon. H, H. LEAVITT, District Judge in the Southern District of Ohio.

COFFIN and MITCHELL, Judge E. NEWTON and HENRY STANBERRY Esqrs., Attorneys for the Plaintiff, and STANLEY MATTHEWS Esq., Attorney for the Defendant.

The following Jury was empanneled and sworn, viz:

| | |
|---|---|
| A. P. RUSSELL, | W. P. HUGHES, |
| JOS. A. LEWIS, | CHARLES H. SARGENT, |
| L. MOUNT, | A. W. ARMSTRONG, |
| ROBERT SIMMS, | THOMAS WEBB, |
| HUGH PATTERSON, | SAMUEL GEYER, |
| M. R. MILLER, | JAMES A. SAMPLE. |

Judge COFFIN, on the part of the Plaintiff, presented the points of his case, and noticed generally, what he expected to produce in proof, of the alleged infringement, and thus addressed the Jury. There are three things claimed in the Patent.

1. The cement,—and here the materials were described.
2. The mixture to keep the teeth in place while the cement is being fused.
3. The uniting of the teeth to the plate by the cement, forming a perfect and continuous gum, there being no need of metallic back plates, though they may be attached if desired.

Is Allen, the inventor of this mode, as is expressed in his patent? Has Dr. H., the defendant, practiced, or assisted others to practice, the same method, or substantially on the same plan? Has he made the compound and practiced the new mode of setting mineral teeth? If he has substantially used the same compound, if it has been applied substantially in the same way, then there has been an infringement.

We want no heavy damages, that point has been waved.

The discovery has already attained a wide spread fame in England and in America. Dr. Hunter, it is true, was experimenting, trying, but trying to out do us. As to the propriety of a professional man like those engaged in Patent Medicines, taking out patents for what are the mere experiences of Physicians, it might be well to throw open ordinary improvements to the public. I agree that it is proper for a physician to give to his profession the benefit of that practice; but when one, by direct study and an entire devotion of his time and thoughts, in pursuit of a favorite theory, attains a valuable end, as in Allen's invention, he should be protected in the fruits of his labors.

STANLEY MATTHEWS Esq, opened for the Defence.

Gentlemen, with this personal controversy of the profession, I shall have nothing to do, I do'nt partake of any of the feeling. The questions are, has A. a patent, and have we infringed? We deny both. The plaintiff has no patent, and if he has, we have made no infringement. If he *is* a professional man, still he has a right to a patent, and if we have infringed, we should be held responsible.

The labor of the brain he acknowledged deserved and should reap a richer "honor arium" than the labor of the hand. The time was, however, when a lawyer could not demand a fee; it has been a disgrace to take a patent for curing diseases. In the experiments alluded to, Dr. H, attained results previous to Dr. A. He claimed for this no exclusive right or merit. Dr. H. published, at the time, elaborate descriptions and minutia of his investigations and discovery. Dr. A. for his labors and results is entitled to no more credit than any body else.

The first answer we make to the plea is as to the patent. We claim that it is void from ambiguity. It is impossible to secure a valuable result by following the exact language of the specification.

The Patent Laws require full, clear and explicit language, and descriptions, such as will be appreciated by the average artisan mind on reading the specifications. The reason of this is, the Government gives the inventor a monopoly for 14 years, and for this consideration he must fully disclose his means, so that should he die, the public would not have the important secret die with him.

In this particular, the patent is defective. 1. The cement, it says, is composed of *any of the known fluxes*. The patentee has never discovered that all known fluxes are capable of rendering this service. 2. He says he arranges the teeth on the plate, in a certain way. That was De La Barre's work, and is the mode and method used by him 35 years ago. It has not been many years since natural teeth were used—then other substances, that of the Hippopotamus.

Latterly, porcelain is used, an invention of the French—a Pottery, of Silex, feldspar, and kaolin clay; it was baked into a tooth body—the same materials, in different proportions, were again vitrified or made into an enamel, a gum that melted at a lower temperature than the composition, and less than would melt or warp the teeth. De Le Barre told how all this was done—his material was porcelain, such as was peculiar to the works of the factories in Paris. Dr. A. is not entitled to the credit for this compound.

On the 19th of April, 1851, he filed his caveat for the invention he here claims ; yet soon after this he gave a German porcelain manufacturer $100 for a formula to make a gum compound.

The patent is void for the omission of certain necessary words, apparently done purposely, with a view to deceive the public, and if such a fact can be proved, the court will enter a *vacetur.*

It will be proved that Dr. A. has made sales with verbal instructions other than those of the specifications, and also by secret circulars. We claim that the mode alleged to be Dr. A's. original invention, was previously used by other Dentists, particularly by Dr. Hunter. We shall exhibit three specimens of similar work made in November and December, 1850, which were sent to the World's Fair, in London, in May, 1851, one year previous to the date of the patent.

Dr. H. at the same time put practical work in the mouths of persons which are good now.

Dr. H. denies the use by him actually of Dr. A's. compound, either in identical ingredients, in proportions or manner of combinations. One is a fusible cement, the other a baked body, not contractible in the fire by heat or fire. Dr. H. puts his into the fire but once. Dr. A.'s cracks, has to be cemented up, and again and again put in and baked until perfected. It is not pretended that of the materials of Dr. A. we have used asbestos. As to the process and mode of application, that will be shown at length in the testimony.

### TRIAL.

This suit was brought on a writ dated Dec. 20th, 1852, and endorsed.

"Suit brought to recover damage for infringement by defendant of plaintiff's patent for "improvement in setting Mineral Teeth,' issued to plaintiff by the United States, and dated Dec. 23d, 1851. Sum claimed to be due $10,000.

Coffin and Mitchell, Plaintiff's Attorneys."

The Plaintiff, by his Attorneys, here offered the patent itself in evidence. A mere *pro forma* objection was made to it by the defence, who claimed that it was really no patent, for the reasons alleged above.

Properly to appreciate the testimony and the merits of the controversy, the following description from the patent is given.

It is dated the 23d day of Dec., 1851, and signed by Alexander H. Stuart, Secretary of the Interior, and countersigned by Thomas Ewbank, Commissioner of Patents.

The following is the Schedule of the Patent:

Be it Known, That I, John Allen, of Cincinnati, in the County of Hamilton, and State of Ohio, have invented a new and useful mode of setting Mineral Teeth on Metallic Plates, and I do hereby declare that the following is a full and exact description of the same:

To enable others skilled in the Dental Art to make and use my discoveries, I will proceed to describe the Composition and mode of application.

The Cement may be formed of any of the known fluxes, combined with Silver, Wedgewood, and Asbestos intermixed, with Gold and Platinum scraps, which forms a metallic union with the plate upon which the teeth are set. The Compound which I prefer is composed of Silex 2 oz., white or flint Glass 2 oz., Borax 1 oz., Wedgewood 1½ oz., Asbestos 2 drms., Feldspar 2 drms., Kaolin Clay 1 drm. This Compound should be intermixed or underlaid upon the plate with Gold and Platinum scraps. The gum color consists of Feldspar ½ oz., white Glass 1 oz., Oxyde of Gold 1½ grs. Moisten, and apply with a brush.

APPLICATION.

I construct my plates and arrange the teeth thereon in the usual way: I then apply the Cement in a plastic state upon the outside, between and around the base of the teeth, so as to form an artificial gum upon the teeth and plate. The teeth and gum are then covered with a mixture of Asbestos and Plaster of Paris, mixed with water and reduced to a plastic state. The teeth being thus covered, the wax is removed from the inside of the teeth, and the Cement is applied thereon, and also upon the plate, so as to fill up all the interstics around the base of the teeth. When the Cement and mixture thus applied become thoroughly dry, the work, so united, is put into a furnace sufficiently heated to fuse the Cement, and immediately after the fusion thereof, it is withdrawn from the furnace and cooled slowly. The plaster mixture is then removed and a gum color applied. The work is again placed in the furnace as before, and when fused is withdrawn and cooled as before, by which means the metallic back plates, solder, and blow-pipe are dispensed with, although back plates may be attached to the teeth if desired. Finally,

What I claim as my invention, and desire to secure by Letters Patent, is a new mode of setting Mineral Teeth on Metallic Plates, by means of a fusible Siliceous Cement, which forms an artificial gum, and which also unites single teeth to each other, and to the plates upon which they are set.

I also claim to be the inventor of said Cement, or Compound, a full and exact description of which is herein given. I also claim the combination of Asbestos with Plaster of Paris for covering the teeth and plates, for the purpose of sustaining them in their proper position while the Cement is being fused. JOHN ALLEN.

Witnesses: Nirum Allen,

Chas. D. Allen.

A large number of lengthy depositions were read on the trial, of course, but an abstract can be given of each.

Deposition of Dr. John S. Clarke, of New Orleans, taken under the act of Congress, Feb. 25, 1854. Have read all the Dental Journals but found no mode so valuable as that under A's patent. De La Barre, a French writer many years ago described a mode of setting mineral teeth, in some respects similar to Dr. A's but it was essentially different and in practice was not found to answer the same purposes.

Cross Examination. Thinks he could make A.'s combination from the directions in the Patent.

Deposition of Benjamin Silliman, Sr., of New Haven, Conn. and Professor of Chemistry in Yale College. Borax is usually kept as an article of commerce, in crude crystals, containing their waters of crystalization. It is kept generally in Apothecary shops, but sometimes in hardware stores. It is not usual to find borax kept for sale, in any other form, calcined borax is generally prepared by those who are going to employ it in the arts. It is done by expelling the water of crystalization by heat, which is very easily done, and then the borax can be melted into a glass. Every mineralogist, or the artist, prepares it for himself in that manner. I have seen and adopted Dr. Allen's improved method &c. and have in my own family a complete set of teeth constructed upon that method, which has given entire satisfaction. I think the method superior to any thing else that I am acquainted with. The knowledge I have of the method is for 2 years and was first obtained from Dr. Samuel Mallett, of New Haven, who constructed for me the set of teeth mentioned above.

The work was entirely new to me and I regard it as an eminently useful invention.

Deposition of Dr. Samuel W. Hazlett, a practicing Dentist since 1844, knows Dr. A.'s method. To those who set teeth, the specifications are clear enough to reproduce results as laid down in the Patent. Platina plates were known and used by dentists prior to the date of Dr. A.'s patent and are mentioned by Desirabode, a French writer, and republished in the American Journal of Dental Science, p.p. 430–431. Thinks if cracks occur in cooling he would know they should be filled up without that instruction being specially given him. Understand "known fluxes" to mean, such as are used by Dentists in Blockwork.

A new method of supplying Artificial Teeth and Gums by Wm. M. Hunter, Dentist. "Is there any thing whereof it may be said, See, this is new?" a pamphlet of 16 pages printed in 1852, was presented and admitted as evidence,—admitted by the Defence with the understanding that the method therein narrated had been "successfully practiced."

Dr. Hunter's Formula, and admitted to be such, was read from the New York Dental Recorder, p. 287, Sept. No. 1852, and from page 67 December No. 1851.

Deposition of Dr. Samuel S. White, (afterwards a witness on the stand,) taken at Philadelphia, 29th Sept., 1854. Aged 32, residence W. Philadelphia, occupation manufacturer of teeth and Dental material, am of the firm of Jones, White and McCurdy. We purchased for $100 of Dr. Hunter, in 1852, a formula for a compound for a continuous artificial gum, which was to be fused on to the teeth and plate, thus uniting them together also without fusion to the plate, by uniting teeth in block form and then attaching to the plate by soldering as in ordinary block teeth. This formula was afterwards published, I think in the October number of the Dental News Letter. The money to be paid was to be conditioned on the maintenance of this suit by Dr. Allen, and was not paid until about the 1st of September, 1854.

Deposition of Dr. Wm, R. Webster, of Richmond, Ind. Applied to Dr. Hunter to learn his formula; he told me he took no students for less than $100, and that need not be paid unless I was satisfied that the mode was new and useful, and that Dr. A. could not sustain his patent.

Deposition of Mrs. Hannah Stephens. Dr. Hunter made a set of teeth for me prior to April, 1852. They were discolored once, and Dr. H. voluntarily offered to take them back, and made me my present set free of cost.

Cross examination—The cause of the exchange was on account of the change of color in the gums or teeth; don't know whether the first set were paid for; my son, I suppose settled for them.

Deposition of Dr. Benj. D. Wheeler. Worked for Dr. A. in 1848; he made sets. I made the mechanical part; made set for Mrs. Bartlett, of Kentucky, and others.

Cross examined—Practical results not secured in the specimen spoken of. Platina proper base, but not so good as gold. Practical result obtained by that of Mrs. Hayne.

The first oral witness introduced was Dr. Seth P. Miller, of Worcester, Mass. Sworn. Knows Dr. A. since the summer of 1853, and am acquainted with the principles said to be set forth in his patent; have practiced on them; have over two hundred in use.

I examined, on last Monday, a set of teeth said to have been made by Dr. A., belonging to Mrs. Heaton, of Mt. Auburn. They have a continuous gum on the outer circle, with plates inside; they have a strong resemblance to Dr. A.'s work. I have made the compound, but not from the specifications purchased from Dr. A. or his reputed agent. I use different combinations or proportions of the same essential ingredients, which I manufacture myself. I use platina plates, and nothing not in the specifications.

I find a difficulty after A.'s formula, in baking and fusing. It is not possible beforehand to define what should be the heat.

As to fluxes, glass is a flux; bottle glass, black bottle glass, borax glass, crown glass, potass are fluxes, borax is one of the best of fluxes, it has to be reduced to a glass. A man competent to make blockwork would understand what kind of flux to use. I would use borax glass—would frit the borax of course. The common glass, white or crown glass. To a question by Judge M'Lean—Would practical dentists understand it to be a suitable flux that would answer? We make a special flux, composed of silex, borax, made to a glass, and potass fusa, or common potash.

As to the state of the art before Dr. A.'s method was published. When I commenced I used single teeth, without gum on a gold plate, then in sections, with back plates riveted and soldered to the plate.

He described minutely the process and peculiarity of the manufacture of block teeth. The continuous gum and plate has the advantage of being stronger, has a fewer number of fissures, nearly approaches nature. I knew of nothing of this kind previous to Dr. A.'s. If a break takes place with this work, it can be filed off or ground down, without damage to the remaining teeth. There is a great advance in that light, a great saving of expense, a life-like look produced—a little zig-zag movement can be produced if necessary. If a tooth be too short it can be articulated in wax and filled up with material. It can't be done so with block work.

In the points of cleanliness, oneness, attaching or fusing to the plate, shutting out any food or foreign matter, patients have by this, experienced great satisfaction—there is durability, it lasts well, stands longer without fracture, makes a more easy and perfect fit, and is easy of repair, blockworkers can't thus unite blocks to the plate—gold plate will not do—platina is used, because it requires so high a heat to melt it.

As to DeLaBarre, I read it 20 years ago, and consider it obsolete so far as I know. By those who use Dr. A's. method and *who are successful,* it is considered an improvement. As yet, not one half of the Profession in New England use it; one reason is, on account of the pendency of this suit—the Patent is not established.

A portion of the Profession, in attempting to use it, have failed, while a portion have been successful—the work has not always been put up properly. I have been successful. Those who have failed, have been of those, who have failed in all methods. There is no more occasion to fail here than in any other system.

*Cross examined.*—Have had no assignment of Allen's patent, or privilege to use his plan—have made the cement, not after his specifications. A flux may be used without borax, silex and pottasa. I always use borax—never have made any without borax. Think there could be; think that carbonate of soda would do. What wedge-wood is; I never could learn what it was—it is the common Apothecaries mortar; but what it is composed of, I can't tell. A block workman, from the specifications alone, would not be able to tell, without experiment, what amount of heat would be requisite.

Gold fuses at 2000 degrees; tested by wedge-wood Pyrometer, Gold alloyed with Copper, 18 to 20 carets, is used for ordinary gold plate. Silver will fuse at a lower temperature.

I have never used in some 200 sets, Dr. Allen's gum material; I made a gum enamel of my own; I sell it; have advertized it.

Here the witness was shown a card of his, "Miller & Sterns." The reason I discontinued the use of Allen's compound is, I put in several that have done well, but from the high heat in the furnace, several sets blistered; I had trouble with the furnace. I have sometimes since had trouble in the application of my heat; but I could return home now and do better, from having obtained a better knowledge as to the application of heat. I use essentially the same materials as Dr. Allen; but I am compelled to acknowledge I do not see any superiority, as to strength or in manipulation of these spoken of in my card, over those made by Dr. Allen.

In using Allen's compound, I put it around the teeth and plate, cover the teeth with asbestos and plaster of Paris, place in muffle, when fused, withdraw it and let it cool—some shrinkage ensues, cracks are filled up. As to the Mrs. Heaton sample—I judge from the external appearance it is the work of Dr. Allen.

Dr. M. W. S. Kendall, of Cincinnati, Sworn.—Am a manufacturer of artificial dentures; have made a number after Dr. Allen's, plan in three years past. Made one piece after De Le Barre's formula; (it was presented, and was quite a ragged, specimen,) as translated by Fitch, page 471. J. M. Lock, mineralogist and chemist, aided me carefully to do the work. The directions were strictly followed; we arranged the teeth, backed them, made the paste, and applied with a knife, dried it exceedingly slow, put it into a muffle, of Philadelphia pattern, the fuel was coke; had my eye on the specimen in the fire, it was in good shape before being fused, it was brought out carefully, cooled, and presented the ragged shape, it now bears. It was contracted about as much as Dr. Allen's; it had several fissures; some are seen yet; any clay heated and cooled will crack, but less as it is manipulated. Made specimens after Allen's formula, and a specimen after Hunter's formula; the difference of result was scarcely perceptible; the experiments resulted in about the same article. I have made over fifty sets after the method of Dr. Allen. Have made work for Dr. Taylor and others. Take the pieces of Allen's and Hunter's work, and except from the color, one cannot be told from the other; might know the compound apart, from the excess of coloring matter in one of them.

I have been in the Pottery business; am not a chemist, I have made my own analysis, the articles are the same nearly, separate and the compounds are the same, I cannot see any difference.

I have given a deposition in this case called by Dr. Hunter. I would have no difficulty in making work from Dr. Allen's specifications.

In making all the nice operation in this department of Dentistry, some failures are made, and experince adds to experetness.

*Cross Examined.*—Have been a Dentist, between two and three years, was instructed by Dr. Taylor; was previously thereto a manufacturer of pottery. As to the gum material in the La Barre specimen, I made a slight deviation, but it was to the advantage of De La Barre. I followed the formula entirely, but in one instance; I put in 1-20 oxide of lead; Dr. Lock weighed the portions. The porcelain paste used, was some I had on hand, made twenty years ago by me; *did not get the proportions or materials out of De La Barre.* Dr. Slack, was for a great portion of the time, also present.

Dr. Hunter's specimen, was made from the formula published in the *N. Y. Dental Recorder*, Sept., 1852. I had a patent right from Dr. Allen, to use his method, and besides the patent, obtained from him, separate written instructions; don't know if I had verbal instructions also. Dr. Allen gave lectures.

Dr. Elijah Slack, of Cincinnati, Sworn.—Am and have been connected with the teaching to schools and colleges, of mathematics and physical science, and in the Laboratory, for forty-five years. There is but one as such, I believe, my senior, in the United States—Prof. Silliman; an other old compeer of mine, D. Hare, has recently deceased. The present progress in chemical science, whatever its progress, hitherto, is greater than ever. I was present when Kendall made up his compound after Dr. Hunter's formula; I weighed them out carefully; noted the weights as well as the materials. Was present during the munipulation, present at each step of progress. I observe and recognize my mark, on the specimen produced.

Platina was scarcely ever used when I commenced my studies; it was known as a metal, it is a noble metal, superior as to resisting heat; it is technically, infusable; has little expansion, or contraction; less than any other I know of. The specimens made by Dr. Kendall, and exhibited here, were made under my supervision, and great care was taken in all the steps of the work. I have examined that made after Dr. Allen's formula, and that after Dr.Hunter's formula and is the same result, and nothing different. I have examined the ingredients and the compounds minutely, put them both together, and my conclusion is that they are as near alike as two and two which make four, and three and one, which make four; nearly identical, as we chemists say.

A skilled person accustomed to fusion of minerals and metal, when he has the specification of a patent, would generally know only by practical experiment. It would be indispensible to know how to fuse on to metallic base, the expansion of the metal and compound must be nearly as possible equal; pure gold will not make good work. I found that platina, was the thing, that's the foundation of the important improvement.

*Cross Examined.*—As to expansion and contraction, all materials vary according to their composition, proportions alter the nature of compounds. The result can't be told *a priori,* when the ingredients have different relative expansions and contractions, it cannot be told except by experiment what will be the expansion and contraction of the compound. There are the same ingredients, but in different proportions, in calomel and in corrosive sublimate; one is a violent poison, the other is a well known medicine. Calomel is not a poison, unless given by *quacks.* Dr. Slack, agrees that the "porcelain paste," of De La Barre, as translated by Fitch, was that of which Mr. De La Barre, made his teeth.

Dr. David Wemyss Jobson, a dental author and litterateur, of London and late of Paris, now of New-York City, Sworn.—Have been a dentist for twenty years in Scotland and in London. I was lecturer, and tried to keep up with the improvments in the art, my researches embrace the whole art, the manufacturing part, as well as the surgical branch.

As to the De La Barre method, that was not adopted by the profession, it was found to be a failure, I knew it to have been a failure in De La Barre's own hands. I met him in Paris, in Oct., 1836, and although he did not take me into his laboratory, he gave me a copy of his book, The French edition was given the witness who said he well understood the language, and translated, "porcelain paste seven parts, etc., *frit perfectly.*"

The method has been found to be a failure, when I first saw him he had given up the practice of it, and when I last saw him, he had abandoned the use of mineral teeth almost entirely. I knew Deserabode, his written work was good, but chiefly a compilation. I knew of Dr. Allen's work, first through his agent in London, Dr. Lawley, I saw Dr. Allen in London, one year ago, and in Washington city, and New-York, three months ago. Am acquainted with Dr. Robinson of London, he is a man of standing; it was he who referred me to Lawley.

I consider this new improvement of the Patentee, as *the greatest improvement ever made in the art; it* certainly *leaves nothing more to be done.*

*Cross Examined*—Porcelain teeth were in use in France, from 1815 to 1820. I saw French work, saw specimens of De La Barre. I think I could recognize the work. There is but little difference between the French and American, and much, between the French and English. Here there was exhibited to the witness a dilapitated specimen of an artificial denture.

This specimen is similar to that made in France, in 1825, the teeth and gum are of one material; this looks, like Audebrand's specimens, whose method was given up as a failure; the surface of the teeth do not appear to be ground down by use; cannot say whether this has been practical work in the mouth. ( The specimen alluded to,as hereafter testified, was that worn for some time by Aaron Burr, and was made for him by De La Barre, when Burr was in Paris.) Here the witness was shown a printed work on dentistry, which he acknowledged was written by him ; a clause in a chapter on the De La Barre work, was pointed to, the witness said that was his opinion of the work twenty years ago, but he has changed his opinion since. De La Barre showed me his work in 1826, or 1828; when I saw him in 1836, it was a decided failure. This specimen, if it appears to have been put up by De La Barre, it was not by plates of this construction. This does not look like De La Barre's work, its more like American teeth.

I was once at the Crystal Palace, in London in 1851, and saw no dental specimens on exhibition. The witness was shown the specimen of Dr. Hunter said to have been on exibition there and said, if I had seen this specimen there, I should have considered it a great improvement. I see no back plates here, ( scraping the plate,) this is galvanized platina. Shown specimens of Dr. Hunter and those of Dr. Allen, the result attained seem to be similar.

*Re-Examined.*—I have seen De La Barre's work in his own hand, his was not similar to this; if such a result had been attained, he would not have abandoned it. The old specimen here shown, looks like silver plate, platina alone was used in France, for miueral teeth, there was none other used for French or American teeth. English teeth were set in gold plate ; double plate was used for strength, pure gold used for solder of platina. The French used back plates called by them, ramparts, they were always set that way. The English suported theirs by a pivot in the eenter of the tooth. Ordinary operators, in my opinion, would know that gold is too fusible for plate to be put in the furnace, the man who did not select platina would indeed be ignorant.

*Re Cross Examined.*—This was exactly the way Dr. De La Barre's specimens, usually were, ( like the ragged specimen made by Dr. Kendall and J. M. Locke.)

Joseph M. Locke, Chemist and Mineralogist of Cincinnati. I made an analyzation of the physical character and component parts of the ingredients of the three formulas of De La Barre, Dr. Allen, and Dr. Hunter, respectively.

De La Barre's formula I found in the 2d edition of Fitch 1835—page 471. Dr. Allen's was from a written and a printed copy of the Schedule of the Patent. Dr. Hunter was from a written and also a printed copy of his formula. First I reduced the formulas to their per centage. The first analysis was the proximate analysis of the several ingredients. To arrive at the ultimate analysis take out the component parts of simple bodies and add them together. For the ultimate analysis of the proximate principles I relied upon the established books of authority, Turner, Brande, Ure, Graham, Faraday, &c.

The following is the result of the *Proximate Analysis:*

| | | |
|---|---|---|
| Silica, | De Labarre, | 46.01 |
| | Allen, | 59.95 |
| | Hunter, | 65.07 |
| Alumina, | De Labarre, | 25.00 |
| | Allen, | 6.07 |
| | Hunter, | 4.09 |
| Lime, | De Labarre, | 7.05 |
| | Allen, | 0.07 |
| | Hunter, | 6.03 |
| Magnesia, | De Labarre, | 0.00 |
| | Allen, | 0.88 |
| | Hunter, | 10.07 |
| Oxide of Iron, | De Labarre, | 0.00 |
| | Allen, | 1.31 |
| | Hunter, | 0.02 |
| Oxide lead, | De Labarre, | 13.00 |
| | Allen, | 12.00 |
| | Hunter, | 00 |
| Potass | De Labarre, | 2.01 |
| | Allen, | 3.08 |
| | Hunter, | 4.01 |
| Soda | De Labarre, | 0.00 |
| | Allen, | 4.04 |
| | Hunter, | 2.01 |
| Borax, | De Labarre, | 00 |
| | Allen, | 9.05 |
| | Hunter, | 4.06 |
| Loss in Allen's | | 37 |
| " " Hunter's | | 65 |

Allen's and Hunter's differ from De Labarre's in Alumina. The Hyd. of Alumina, when first heated, contracts a great deal in cooling. In order to ascertain the relative contractility of the three specimens, I have them arranged on this card, the original shape before they went into the fire is marked and their present shape upon the card shows the amount of shrinkage. To measure it exactly, I have here a guage, in the groove of which the specimen is placed when heated and after its cooling and the minute divisions on the scale mark accurately the difference. The amount of contractility of the two specimens made by Dr. Allen and by Dr. Hunter's formula, are near alike, being but seven-ten thousandths in favor of

Dr. Hunters. As to the ragged specimen of De La Barre's I think no mistake was made by me. None were observed during the process.

The compound went into the muffle, perfect, I saw it while in, in good shape, just before the point of vitrification. When Dr. Kendall saw it softening he drew it out. The degree of heat obtained was indicated to be, by Daniel's Pyrometer 3761 degrees Farenheit, 300 degrees above melting point of good soft iron. The other compounds Allen's and Hunter's fused at 2264, just above the point of fusion of pure gold. I think the De Labarre formula was substantially followed, American Porcelain paste was substituted, but that I esteem to have been advantageous, for if the French Porcelain had been used, it would have required a higher temperature, which perhaps could not have been reached.

The result of the ultimate analysis as to Alumina, Silex and the flux, of the Allen and Hunter formulas show that their contractility was about the same—all the physical characteristics were about the same. The excess of the Hydrate or water in the Alumina in De Labarre's was so great that when the flux vitrified it produced a sudden drawing up and did not fill up the pores and fit to the plate and so warped the plate that it would not again fit the matrix, the shape of the mouth, in which it was fashioned. In Porcelain statuetts in France, the models have to be larger, to secure requisite size, after shrinkage and are not distorted as they shrink all around, but it is more difficult where it has a metal attachment.

I am decidedly of the opinion that subsequent experiments, after the formula of De Labarre, would produce the same results, work that would not suit in the mouth.

*Cross Examination.*—When identity is sought, it may be established in different ways—there is a chemical identity. The action of an overshot and an undershot wheel may produce similar results by different processes. It does not follow that ingredients being chemically identical, that they are mechanically identical. The proportions of the materials may vary the results. The same ingredients in greater proportions would vary the results. There may be an essential difference in the result, in the union of simples into one compound and the union of the same simples in different proportions into two compounds, and the union of those two compounds into one compound—Asbestos for instance is composed of lime, magnesia, alumina, and potash, you can't take those articles and make asbestos by compounding them. Mr. Locke was closely examined on similar points, by way of cross examination. The specimen of Dr. Hunter was whiter than that of Dr. Allen.

The witness was shown three specimens of artificial Dentures, and said, he did not believe that the result thus shown could have been attained by De Labarres' formula. (One of them was made after De Labarres formula, by Dr. Geo. W. Kendall.)

The depositions of Drs. William H. Davidson and Edward L. Roberts of New York City were read; both had tried to make work after the Fitch translated formula of De Labarre, but failed.

This closed the testimony for the Plaintiff, his Counsel announcing, that they had a large mass of testimony still to offer but would reserve its introduction, as rebutting evidence. The examination for the Defence commenced on Saturday afternoon at 2 P. M.

### TESTIMONY FOR THE DEFENCE.

Saturday, 2 P. M. May 12th, 1855.

Dr. Harris R. Smith, sworn.—Have been a Dentist between 11 and 12 years; I now reside in Terre Haute, Indiana, was here for four months in the Ohio Dental College. I succeeded Dr. Allen as Instructor there; am acquainted with his mode, by written instruction and recipes, as also by oral instruction. I have put up some work under these instructions, some of the work does well and some does not, as to its general utility I have not had full time to test it. I have found it to have failed more than other work; it appeared to be good when done, but did not in all cases prove very well; in the majority of cases it has not come up to my expectations. On reading the specifications would not understand that the borax must be fritted, unless it was so expressed in the specification, at first it would not be fit for practical use in the mouth. He described the process of baking and applying the gum color.

*Cross Examined.*—One year ago, last winter I worked in mechanical dentistry, some of the work endured well, others not. Perhaps I was not expert, perhaps the materials were not perfect; care and skill, of course are necessary to success. Borax and potash are known by Dentist as fluxes, black bottle glass for flux, any one would know, would not do. The work on account of shrinkage had to be filled up and placed in the furnace several times after. Few persons know beforehand the extent of heat required. I did not know except by experiment and examination.

*Re-Examination.*—Did not know before instructed, what heat was required; by "plates constructed in the usual way" I should understand silver or gold plates, particularly gold.

*Re-Cross-Examination.*—All persons should know that some of the materials require for fusion, a higher heat than gold.

Dr. Samuel S. White (His Deposition was read previously by the Plaintiff.) My residence, boy and man for 19 years has been in Philadelphia; am of the house of Jones, White & McCurdy, and manufacture and sell to the profession Mineral teeth, perhaps $\frac{4}{5}$ths of the teeth made in the United States. I know both of the parties to this suit. I have known Dr. Hunter since 1847–8. I conversed with him years ago, as to continuous gum work. In the fall of 1849 Dr. H. was at Philadelphia and told me, I can't give the language substantially, that he had been experimenting to obtain a compound that would fuse and unite in a plate forming a whole half denture, with but little contraction, and that as to his success at that time, he then said, he had arrived at a point when he could make a compound that would not materially shrink.

I know Dr. Allen; I purchased under his Patent, the privilege to use his Cement. He gave me, beside the specifications, instructions on a sheet of letter paper and the choice of two formulas—they were different; understood the latter to be his "improvement." The plan pursued by myself, is not that of Dr. Allen's but is a secret of my own, having experimented to make the best. I never put up many on Allen's or Hunter's plan; Allen's contracted more, how much I did not measure. I preferred my own modification. The fluxes used before 1851 were glasses; borax was named in the books, but not used. I would not know, from reading the specification, which article of borax was designed to be used; there would, in the use of different kinds, be much difference in the fusibility.

The first piece of an entire half denture of continuous gum work I ever saw, was in the latter part of 1846 or 1847, in New York city, apparently made with French teeth, it was a large mass, a compound of continuous gum on a base ; it was represented to have been worn by Aaron Burr, and to have been made by De La Barre. This specimen (the one referred to before) I believe is the same one, it has been much mutilated since I saw it, then there were but two or three teeth broken from it. It was then in the possession of Mr. Jones, at present, one of my partners.

Dr. Hunter's formula is better to prevent contraction, as he sieves out part of the granules, and thus makes what he terms a granulated body. I observe the same method; but my manipulations are after my own particular views. If the granules of Dr. H.'s compound were reduced to powder, it would contract more. Dr. A. does not provide for this granulation. Porcelain in this country fuses at different degrees of heat, according to its composition. I did not know of platina being used for dental plates until I saw the specimen of De La Barre's in '46. If it had been in use, I should have known it; with present formulas gold plate could not be used. The alumina of commerce differs, it is not always of the same purity, and not equally suitable for this kind of work, and some would contract more than others. The borax of commerce is not always of the same purity ; my knowledge of block work would have taught me to have driven off the water of crystalization from the borax, 1 oz. of which is prescribed in the specification. Previous to a secret receipt I bought of Dr. Wildman, I would not have known that borax should be "fritted."

*Cross Examined.*—The receipt mentioned, was purchased of Dr. Wildman in 1849.

Knowing that Dr. Hunter was experimenting on this important subject of continuous gum work, I solicited the sale of the benefits of his experiments, for my use as a manufacturer to supply the dental world.—We met several times and conversed on the subject. I finally wrote him a letter, urging him to impart the desired informa tion; he soon came on—we were together in my Laboratory and agreed on the price for the receipt and information which he proceeded to give me—(letter presented which he had written—recognized by the witness.) The first letter I wrote was on the 1st of March, 1850; H. came to Philadelphia, to the best of my recollection, in the fall of 1850; the receipt he gave me, (it has no date,) I have at home, in his hand-writing; I was to give him $100, and as we had a running account with him, I did not make a final settlement, including the pay of $100, until September, 1854; I did not expect to be called here; was in the city here attending the Dental Convention; I can give the ingredients, for it was afterwards, in 1852, published in the Dental News Letter; the written one was received some time before its publication; I obtained it, to put a brother of mine to work, not expecting to apply it myself in making continuous gum work. We sell platina for plates; do'nt make the plates; platina pins, it is evident, were used in block work to stand heat. Borax, as a flux, was a new thing to me as stated above.

*Re-Examination.*—The Catalogue shown is one issued by our house, and contains a list of Dental Supplies.

Dr. Alexander A. Semple.—Reside in Steubenville, Ohio, am a Dentist of 18 years practice. This is the written assignment (June, 1853,) and this the written formula, with directions furnished to me by Dr. A. when I purchased a right from him under his patent. I received also oral instruction, and Dr. A. demonstrated the work by practical operation that I might do the work. I used Dr. A.'s materials, but in 10 or 12 cases did not succeed at all. I was particular in observing the instructions; the work pleased me when it came out of the furnace, it looked well. Work accumulated that generally gave dissatisfaction, and in several cases fell to pieces, and had to be done over again.

*Cross-Examined.*—I put my work up to order; the first piece I attempted was a double denture for a lady in Pittsburgh; I had never done block work; I commenced with the upper part first; in three months after the lady got her teeth, they were sent back to me—part of the body and color in both upper and lower set had scaled off—fractured no doubt from ordinary use. I have knowledge, particularly, of but two specimens, all the rest, I learned, failed in the same way; there were at least fifty fissures in the one yet in a lady's mouth in Steubenville. I have not paid Dr. A.; I refused before six months, when it came due, on account of these failures, and have not experimented on the method since.

*Re-Examination.*—I refused to pay, because I felt that I had been imposed upon by the Patent. The reason of failure, I think was the want of strength; I followed carefully the instructions of Dr. A.

Deposition of Charles Steemer.—Residence, Cincinnati, Ohio; am an enameler of metals; in May, 1851, I sold to Dr. A. a formula for enamel for $100, and for $30 a preparation of gum color.

| | |
|---|---|
| 2 oz. | Silex, |
| 1 oz. | Borax, |
| $\frac{1}{8}$ oz. | Feldspar. |
| $3\frac{1}{8}$ oz. | White glass. |

I prepared a crucible of the compound for him, and made the experiment before him, of placing it on the plate; Dr. A. furnished the platina plate; I had a patent for enameling on cast iron. I also sold, two years after, the same receipt to Dr. Darling.

*Cross-Examination,* taken at another time.—The witness not understanding the English language, Leopold Burckhardt was duly sworn to act as interpreter. Dr. A. came to me and asked me whether I could make an enamel of that kind, that would flow upon platina plate, and I answered him that I would try, and did so. Dr. A. came to me with teeth put on a plate, and lined with plaster of paris inside and then put the enamel I had made, on the teeth in my presence; this was the first work of the kind I ever saw done. The article in the Dental Recorder, p. 271–2, Aug., 1852, was signed by me, but I did not then understand its full import as it is now explained to me; Dr. A. gave me his word and honor he did not use the article which I taught him, and if I signed this article (which I did not understand at all) he, Dr. A., would give me employment, but he then went on to New York, and I have not seen him since, until a short time ago; it was a voluntary act of mine; I do not recollect of ever having told Dr. A. that I had done wrong, and was sorry for what I had done.

*Question.*—Did you not state to Dr. A. or Mr. C. Burckhardt, that you had been induced to do what you did, that is, to publish a card to the Dental Profession in the United States of America, proposing to give instructions to sell formulas, &c., to the profession for doing this kind of work; and raising your hand, did you not state that they told you, that if you would do it, that is, come out and claim it as your own, that it would be worth the half of California to you.

*Answer.*—I do not recollect that I have ever said so, either to Dr. A. or Mr. Burckhardt; nor do I recollect ever to have written such a card, but I have manufactured some stuff after my formula, and left it at Mr. Denhard's store to sell.—(Defendant objected to this.)

Deposition of Mr. Charles F. Thomin. Charles Steemer told me of his prospective sale of his enamel to Dr. Allen. On the 20th of June, 1851, Dr. A. got me to get Steemer to sign a publication correcting a former published statement, and authorized me to say to Steemer that he would give him $200 for doing so. Dr. A. experimented in my furnace, as early I believe, as 1848, for making continuous gum-work. Steemer instructed one man to my knowledge, in putting teeth on plate.

The following certified copy of the *Caveat* was presented and admitted as testimony.

*To the Commissioner of Patents:*

The petition of John Allen, of Cincinnati, in the County of Hamilton and State of Ohio.

Represents, respectfully, that he has made certain improvements in Dental Surgery, and that he is now engaged in making experiments for the purpose of perfecting the same, preparatory to his applying for Letter's Patent therefor.

He therefore prays that the subjoined description of his invention may be filed as a caveat in the confidential archives of the Patent Office, agreeably to the provisions of the Act of Congress, in that case made and provided.

He having paid twenty dollars into the Treasury of the United States, and otherwise complied with the requirements of the said act. JOHN ALLEN.

Cincinnati, April 7th, 1851.

What I claim as my invention, is a fusible cement, of which an artificial gum is formed, applicable to artificial teeth, and by means of which they are set. The mode of applying the cement is as follows; when the teeth and plate are properly arranged upon a cast of Plaster of Paris, and the teeth covered with the plaster, I use the cement, under, between and around the teeth. This cement is composed of silex, oxide of tin, oxide of lead, manganese, potash, and wedgewood. The teeth being thus arranged, are put into a furnace and the cement is fused, the cast is then removed, and when cooled, the outside plaster is taken off. I then use a preparation of the oxide of gold, so combined with the cement, and so applied, as to produce, when finished, a true gum color; again the cast is put into the furnace until the latter preparation flows, it is then removed, cooled slowly, and is ready for the mouth. JOHN ALLEN.

Filed, April 29th, 1851.

Deposition of DR. EMORY G. DARLING, Dentist of Cincinnati. Charles Steemer sold to me in July, 1851, a receipt for his enamel.

MONDAY MORNING.

MRS. ELIZA W. GUILFORD, Widow of Nathan Guilford.—Testified, that the full set of teeth which she now wore, were made for her by Dr. Wm. M. Hunter, the Defendant; the cast for which was made in December, 1850, or January, 1851, and they were placed in her mouth in the latter part of May, or the first part of June, 1851. They have proved to be as good in service as natural teeth, and a little better, for they neither decay or ache. They have been in constant use; she can eat corn off the cob, or bite a cracker without injury or experiencing any disadvantage. Having retired, the teeth were sent in for the examination of court and jury, and proved to be continuous gum work, fused on platina plate.

Deposition of Dr. Elias Wildman.—I am 43 years of age (this 26th day of August, 1854;) reside No. 8, North Eleventh Street, City of Philadelphia; occupation, a Dentist; I commenced the Dental business in the spring of 1836; am acquainted with the parties to this suit; I am familiar with the French language, and translate it. The article cited below is a correct translation of the spirit and meaning of the author, Delabarre, and is contained in pamphlet marked B, page 145—149 of Vol. 6 No. 3, April, 1853, the Dental News Letter.

(This article was translated by Dr. F. Badarague, of Va., and corrected for the News Letter, by Dr. E. Wildman, of Philadelphia.)

"The following composition embraces all the conditions required. Porcelain paste 7 parts; calcined gypsum 1; white sand (quartz) 1-20 of the mass; such oxide as you desire; 150 grains per killo gramme; grind perfectly fine."

1. I consider that the published instructions of Delabarre are clear enough to enable a practical manufacturer of teeth to produce the continuous base according to Delabarre's method.

*Question.*—Theoretically considered, do you believe the uniting of teeth already baked to a gum-plate by means of stays or pivots, and a material that will fuse at a less heat than that required to vitrify the tooth as being identical in principle of construction with other formulas that may only require a less heat to accomplish the same end?

*Answer.*—If it means that fusing at different grades of heat which shall be less than the fusing point of the teeth, I consider the principle the same. C. F. Delabarre's work which contains his method was published in 1820.

Dr. Jobson asked to make a slight correction of his previous translation of the French word he had translated "to frit," "*broyez*," meant as he first supposed, to rattle, to make a noise,—it means to grind, to mix up.

Deposition of Dr. Charles B. Foster, Dentist of Philadelphia. I bought a right to make A.'s compound, from his agent, B. F. Smith, and in a few days after received the right, signed by Allen himself. I received also special written instruction, copy attached to deposition, in which is language, "first form a frit in the following manner, &c.." this is different instruction from that first given; does not use the A. formula, for he has made his own improvement on it.

Deposition of Dr. Lawrence W. Bristol, Lockport, N. Y.—Fifteen years ago, saw a specimen of continuous gum work manufactured by Dr. Thomas Harrison, then my partner; it was made for George W. Darling, of that county, and worn for 7 or 8 years. The specimens were exhibited to the court and jury. Similar work had been made by him, and worn in Canada West, St. Catharines; and in Buffalo, N. Y. A specimen was worn by Henry Harvey, President of Bank, Lockport; 18 years ago I read Fitch's Translation of Delabarre.

Deposition of Dr. THOMAS HARRISON, of Buffalo, N. Y.—Dentist for 30 years, now 63 years of age; twelve years ago made a set of teeth for M. Darling, a tavern keeper; he wore it one year; I invented a paste, and enameled it with gum enamel; I commenced experimenting in 1834; some of my work was practical and used. Before making a specimen sent herewith, I discovered a failure in the coloring, and remedied it.

*Cross-Examined.*—Never published my results; the great trouble was to prevent warping in my way.

Deposition of PALMER BABCOCK, New York City.—Dentist; Dr. A. made an experiment in my house, and in one case the compound was fused eleven times.

Deposition of SANFORD BABCOCK.—I was in employ of Dr. Barlow from 6th of March, 1852, to 12th March, 1853. He had a right from Dr. A. The work made by them had to be put into the fire from 3 to 5 times, and was not of practical utility in seven-eighths of the cases; does not consider the patent specifications to be practicable; teeth to be set well he thinks should have backing; he recounted several instances of failures by Dr. Barlow.

Deposition of DANIEL H. PORTER, New York City.—Am a manufacturer of mineral teeth; I bought a right of Dr. A., the formula was not the same as is in the patent; I was constituted his agent and sold one right.

To "frit" is necessary, and that is not provided in the patent; the composition is changed by fritting, which is done by heating in crucibles until the mass is melted, to get off the water. I am interested in the patent being declared null and void, for I would thereby be permitted to enlarge my business. I am bound to protect those sold for Dr. A.

Deposition of JONAS W. SMITH, Brooklyn, N. Y.—Was partner with Martin K. Bridges, as Dentists; we sold Dr. A.'s rights; the work done by A.'s formula failed, and we have abandoned his composition for more than a year, and have taken that of Dr. Miller, of Worcester.

Deposition of STEPHEN D. WILSON, of New York.—Have a set of seven mineral teeth worn by me, made by Beers & Morgan.

Deposition of GEORGE E. HAWS, of New York City.—Been a Dentist since 1824; has a right from A.; favorable to his method; it answers a good purpose, but saw work like it, but inferior, before A.'s was introduced.

Deposition of GEORGE SMILEY; New York City.—Saw the specimens of Aaron Burr's teeth, exhibited in 1841–2; they were brought to the city by Dr. Graham, who then said they had been used by Aaron Burr; the broken teeth were then on.

Dr. Charles C. Allen, by deposition testified, that he saw the Burr set in '44 or '45, with the teeth then attached.

Oral Witness, Dr. Wm. H. King.—Have been a Dentist since 1848; formerly of Cincinnati, now of Pittsburgh; I knew Dr. Hunter in 1849; saw a set of teeth with continuous gum in 1850, made by him; there were two upper sets, one of them Dr. H. showed to me as his "improvement," in the Dental Furnishing Store of Dr. J. M. Brown, in the first part of November, 1850; they arrested my attention, and I tried their strength by pulling the teeth with my fingers; there was gum on each side of the base of the teeth, and different from any that I had seen; I judged both specimens to be single teeth; one was united to the plate by the cement alone; the other had back straps also. I am satisfied as to the date, as I recommenced my Demonstrations in the Dental College in 1850–1; and Dr. H. said, I ought to be able to demonstrate the method. I communicated what I had seen to Dr. Taylor; soon after, Dr. T. brought me the first sample I had seen of Dr. A.'s enamel work; it was called "Levett's Enamel," and would scale off; this was, as I stated above, after Dr. H. showed me the two specimens adverted to, and which he announced, were prepared for exhibition at the World's Fair. In regard to the Levett's Enamel, Dr. Taylor said, he, Allen, had bought the right for Hamilton County.

*Cross-Examination.*—The place at which Dr. Hunter showed me the two specimens, at the time related, was in the Dental Depot of Dr. J. M. Brown, N. W. Corner of Fourth and Walnut Sts., Cincinnati, Ohio; Dr. Brown and John Toland were also, I believe, present.

He showed me two upper sets, one on gold plate, and the other on platina; the gold plate had gum on one side, the other had gum on both; I do'nt know what was the compound or method used; I had known Dr. A. when meeting him, since 1848; but never was in his Laboratory, and knew nothing of his making experiments until I was shown the enameled specimen of his, by Dr. Taylor; this was a platina plate enameled, but no teeth attached.

John G. Toland.—Have been engaged for 6 years in the Dental Depot of Dr. Brown, just alluded to; was there in the summer of 1850; at which time Dr. H. purchased of us some single teeth; he soon after showed in the store, some specimens of artificial teeth he had prepared to send to the World's Fair, Crystal Palace, London. There were three sets of similar styles, had all the general appearance of these now exhibited to me. They were shown some time before the sailing of the U. S. Vessel taking American products and manufactures to the Great Exhibition—say three months before—Dr. H. bought his materials of us several weeks before the specimens were produced. Dr. King was in front of the counter at one time when Dr. Hunter was behind exhibiting what he had accomplished, and Dr. King was called back to witness also what Dr. H. had done. I subsequently conversed with Dr. A. as to his experiments; this was in warm weather I know, for the doors were open, say June, 1851; he bought specimens of continuous gum-work, and said he had never got anything to please him so well as that; it was a piece of continuous gum-work on platina; no backings, if any, they were the few back teeth; I saw the platina pins in the teeth through the body; he was in often, sometimes daily, afterwards, his office being nearly opposite, where Dr. Taylor's is now.

*Cross-Examined.*—Dr. H. showed me the 3 specimens at one time; one was full, the other partial, of upper jaw, double joined by springs; he afterwards told me he had sent them to the World's Fair; he subsequently told me one of the boxes miscarried, and was returned to him in a damaged condition; has shown me work all perfect; I did not examine it minutely, but its general appearance was perfect.

E. Penrose Jones.—Had been intimate with Dr. H. for years; knew of his experimenting, and saw the specimens he sent to the World's Fair; saw them when finished, before they were shipped; they were, I believe, this new method of continuous gum-work.

Dr. Henry Crane.—Was a Dentist as early as 1832, in Hartford, Conn., for some years last past in Cincinnati, since 1840. Have known Dr. H. for 12 years; have known of his experiments on continuous gum-work since 1847; when I met him on the corner of Fourth and Walnut Streets, and talked with him on the subject. He told me what he already had arrived at, and I think he said he had made partial sets; I first saw whole sets in 1850; then he had prepared two or three specimens for the World's Fair; I recognize two of these is of that number; thinks he saw the specimens in November, 1850. The one on a silver plate galvanized, he said, was on a new plan; as a Dentist, he had used crude borax as a solder.

*Cross-Examined.*—I recognize the set before me, not from any private mark made, but from the chamber and peculiar style of the work; I think both were made on the same principle; I cannot tell if the union is the same, for one is banded and hides the joint; the gold plate of course, is united after the fusion; the alteration was to the shrinkage of the arch.

Edward Kinsey, Silversmith of this city.—Was at World's Fair in 1851; saw Dr. H.'s specimens of Dentistry there; one had a tooth broken out; I am not certain, but think the upper and lower circle were united together by a spiral spring; I saw the specimen in Cincinnati before it was shipped over to London; I also saw it at Dr. H.'s when it came back; I was in the Crystal Palace 14 days, and saw the dental specimens every day.

*Cross-Examined.*—Saw no one in special charge of the teeth; the case which with glass side encased them was fastened, and had to be left off to see the specimens; the tooth broken was the eye tooth of the upper jaw, (it has been refilled, as by minute examination it may he discovered) I suppose it was broken in a test of the strength of the teeth; it was broken off next above the gum; I think there were three sets there.

There was presented as evidence, the notice by M. Sullivant, Ohio Commissioner of a place assigned for Exhibition, and one from President Fillmore of the result of the Exhibition.

Dr. Hardy, formerly of Boston, now of Cincinnati. In the fall or winter of 1850 or 1851, I had an interview with Dr. H., who said he had a set to go to the World's Fair. I set or mount block teeth, and coming from Boston I recollect my surprise at finding that consummated out West which we of the East had so long desired to accomplish, to find that so easy here which was so difficult there, as to the shrinkage of the arch.

*Cross-Examined.*—There was of the specimens seen by him one piece and an upper set, on a plate; he was shown some specimens of continuous gum-work, which he said looked like that shown him by Dr. H. as spoken of above.

John G. Jones.—Was in Dr. H.'s Laboratory in 1848, and in '49, the season of the Cholera, and saw Dr. H. experimenting on continuous gum-work; saw the specimens he made and sent to the World's Fair.

Dr. A. M. Leslie.—I was connected with the Ohio Dental College as Professor of Mechanical Dentistry. In 1846 I visited the laboratory of Dr. Hunter, who was then experimenting to overcome the shrinkage of block-work. I doubted his success and on reasoning with him, he said he would use teeth already manufactured, which had already shrunk, the compound a silicious body, uniting the teeth to the plate. His experiments were continued until crowned with success. The first perfect work that I saw was previous to the World's Fair; there were two sets, an upper and an under one, united by springs, and this specimen (one exhibited to him) looks like it; it was just such a piece of work. I attended, at Louisville, in Sept., 1851, the meeting of the Mississippi Valley Dental Association, the proceedings of which I reported in the American Journal of Dental Science. Dr. Allen exhibited to the Association, specimens of what he called improved plate work; they were upper arches; what he claimed peculiarly as his, was the uniting of teeth to the plate, without intervention of solder or back plates. He did not claim, as original, the representation of natural gum. I claimed at that meeting for Dr. Hunter, success in the achievement of the whole matter. My opinion is, that no practical result can be attained by Dentures without back plates; work after

Dr. Allen's patent will not stand, as they have not stood. This is a specimen furnished by Dr. A. in 1849, to show my Dental Class. I presume it is brass; it is gilt work; the teeth are united with silver solder, and then gum flown over it; he then did not claim greater success. I will test it by nitric acid—(after testing it) this is brass. My experience is that Dr. H.'s will stand, and that Dr. A.'s is good for nothing. In 1846, Dr. H. stated to me of his experiments and what he was trying to accomplish; he did accomplish what he designed, in the specimens I saw before they were sent to the World's Fair. This work (a handsome and regular specimen) was put up April 28th, 1855, by George W. Kendall and myself, after the formula of Delabarre, as found translated by Fitch. It has preserved its shape so far as shrinkage is concerned, and no doubt would prove practical work in the mouth.

*Cross-Examined.*—The Delabarre specimen spoken of is soldered with fine gold on a surface of platina plate; Mr. Kendall made the manipulations; there was but one slight fissure observed after the first vitrification, which was filled up, and the only other baking given was when the work was re-introduced into the muffle for fusing the gum color. The porcelain body we made ourselves ; this paste was not exactly as the Delabarre formula, I think it was an improvement on his; we put no alumina in the body ; there is one part in eleven, of alumina in the paste. (The witness was quite sharply searched on the subject of the composition of the paste, and as to alumina.) I used the compound we did, because I thought it would make better work than porcelain. I answer I have not been an active partisan against Dr. A. I took grounds against the validity of his claims set up in the Association, and my report was a minority one, signed only by myself; the majority report was signed by Drs. Goddard and Taylor ; it was different from mine. I did speak of Steemer, and gave the facts in the case; it is true I was the only one who voted for my report; the majority report received the sanction of and was adopted by the Association.

*Re-Examination.*—A resolution was offered by the Association to award a gold medal to Dr. Allen (which was not acted upon) for his improvement. The foundation for the report that Dr. A. offered to pay for it himself, was, his offer made to me in private beforehand, that if the Society hesitated on the score of expense, he would pay for it himself. The main ingredients of the porcelain paste we used in the Delabarre specimen, were silex and feldspar, same as in the body of the tooth ; the French tooth contains a large amount of alumina ; the ultimate analysis of the paste is the same as that of Delabarre's ; porcelain has 6 parts feldspar, 4 parts silex, and 1 part kaolin,—11.

*Re-Cross-Examination.*—The French have two articles of porcelain paste, one is tender and soft, the other hard. In Sevres china there is not one-fourth alumina; the manner in which Dr. A. proposed to pay for the gold medal, was, he said that the Society was poorly able to be at the expense themselves, and he would pay for it himself.

Dr. John Locke, Sr.—Professor of Chemistry for a number of years, in the Medical College of Ohio. My personal acquaintance with Dr. Hunter has been almost continuous since 1849 ; as early as 1844, Dr. Jesse W. Cook, who was at that time a partner of Dr. Hunter, came to my laboratory and conversed upon the subject of artificial dentures, and particularly as to the shrinkage of the arches. He said they were experimenting to overcome the shrinkage of the continuous gum-work; in 1849 or 1850, Dr. Hunter himself sought me, and we conversed on the subject; I made several suggestions as far as chemistry related to the object of which he was in pursuit. In 1850, previous to the World's Fair, he showed me specimens secured by a material whose contractility was near to that of platina; they were complete sets, the gum continuous, the teeth cemented to the plate by semi-fusion ; he was confirmed in his remembrance of the facts stated, by the presentation to him before the court, of a letter written by himself to Dr. Hunter, dated October 18th, 1851. The letter says, that he, Hunter, had made for himself, (Dr. Locke) a set of continuous gum-work, temporary teeth, one year previous, and a perfect set for a gentleman since. The satisfactory test of the similar contractility of Dr. H.'s compound, might be better appreciated by the Jury, when he stated, that the platina rivets exactly fitted the holes, after the exposure to the heat, as they had previous thereto; in an experiment made in my presence; the plate was platina.

*Cross-Examination.*—What Dr. H. was engaged at in his experiments, was to overcome the shrinkage of the material to be used for teeth. The object was to extend the porcelain work beyond "block-work." In October, the temporary teeth were fitted and the continuous gum-teeth were in hand ; it was but a short time before the teeth were completed which I now wear; it is evident in this material or compound of Dr. H.'s its contractility and that of platina are about the same.

*Re-Examined.*—The teeth made for me were once repaired ; I accidently let them fall, and one of the teeth was broken off, but it was speedily and happily repaired, perhaps improved.

Dr. Taliaferro.—I have known Dr. H. in this city, for 8 or 10 years; he made a set of artificial teeth for me first in 1848 ; I heard him speak in 1847–8 of his experiments ; they were called I believe, block-work, with gum representation.

The Dr. exhibited the dentures thus made, and the specimen was examined by the jury and judges. Dr. Taliaferro said, the teeth had answered a most excellent purpose; he could chew anything almost; they had been slightly repaired once or twice, to remedy injuries by falls ; in 1849, Dr. H. made him an alternate set.

*Cross-Examined.*—There was no repairs done to the set made in 1848, other than to apply a new portion to a tooth broken off; a similar casualty happened to the set made in 1849, and it was also repaired; these teeth submitted to you, are solid body of teeth. Dr. H. had not my teeth long for repairs; the other set I have at home, has the gum color applied both inside and out, like the specimen sent to the World's Fair, here exhibited.

*Re-Examination.*—The teeth I wear, and those at home, are precisely alike.

E. S. Wayne, Chemist and Druggist.—I have known Dr. H. 10 years; I know of his early experiments, perhaps as early as 1846, certainly in 1847, for I made a preparation for him; his principal object was to prevent the shrinkage in the fire, of the cement, which was to unite the teeth with each other and to the plate. On the first of December, 1850, I first saw perfect specimens made by him; they were two full sets and one on ——— plate; the preparation I had made for him was pure alumina. He was shown a pamphlet; this is a catalogue of established chemists, stating what wares are to be had at their warehouses; of borax, there is but one article of commerce—the others are subsequent preparations. I am a practical chemist, and would dry and frit borax for the use assigned, but if fritting had been intended by the Patentee, he should have directed "fritting" to be done—if meant, it should have been stated. The design Hunter had, as he expressed it, was to make a perfect arch, joined to the plate.

Dr. Charles A. Lane, of New York City.—I have been a Dentist for 14 years; I first became acquainted with the operation of continuous gum-work in 1845; which was the work of Dr. Jonathan Dodge, now deceased; he described the work; it was nearly as possible the ordinary gum-work, around what appeared to be single teeth set on platina plate; from their appearance, I could not tell how they were united, whether by linings or not; but evidently it was a gum body, uniting the teeth together and to the plate; I do not see any particular difference in appearance between this specimen (the one made by Dr. Hunter and exhibited at the World's Fair) and those; he styled it continuous gum-work; I saw two specimens, one was an entire upper set, the other was a small piece containing about a half-dozen teeth; I was but a casual visitor in his office.

*Cross Examined.*—Platina plate at that time, for such work was no new thing. I know no more of the compound than what little Dodge told me at the time; I do not know, except from their appearance, that they were made for practical use. Dodge died 18 months afterwards.

*Re-Examination.*—There was no other visible means by which the teeth were united to the plate, except by the gum.

Dr. Samuel Wardle.—I have heretofore made a deposition in this case at the instigation of Dr. Allen. I have had practical experience, as a block workman, for eleven years, on the 3d of June next. I was called upon by Dr. Allen to make a specimen, and this is it. I was called upon to put a body around the teeth and fuse it like blockwork. I prepared the materials according to the formula of Dr. A.'s patent. I took the glass of borax, pounded it up dry, then wet it, and applied the compound and fused it. There were some cracks observed, which were filled up. I put the gum color on it and then again applied the heat. In 1830, I became acquainted with Dr. Wildman's secret recipe. Previous to that time I had used no other flux but plate glass, I had not used borax. Then for the first time, in the language of Wildman's formula, I became acquainted with the use of "glass of borax." Had it not been for this experience, I should have used the borax of commerce. Fritting is not always done. It is specially laid down when it is expected to be done. If it be not said to be necessary I do not do it. I made this specimen after the formula of Delabarre, I followed the translation by Fitch. I took for block body my own composition. There is no more trouble in making mine than Dr. Allen's. It is composed of feldspar 6 parts, silex 1½, and kaolin ½—Wildman's.

*Cross Examined.*—I obtained Wildman's receipt in 1850; its merit was in its gum color. The only thing in which I varied from the specifications in making a specimen on Allen's formula, was in the amount of borax. I did not take 1 oz. The reason was, on my first experiment, I took one ounce crude borax and I made miserable work. In the successful specimen, the compound contained one quarter of an ounce of the glass of borax. This was made in March, 1854.

Deposition of Dr. Joseph Doughty, New Paris, O. My knowledge of the Allen work was gained from my experience as a Mechanical Dentist in 1852, with Dr. P. Knowlton. Work made after the Allen formula, scaled off, and Dr. Knowlton was for a while, discouraged at the want of success by that method. Allen gave the Doctor afterwards additional instruction.

The witness was closely cross examined as to the furnace work.

Deposition of Dr. Joseph G. Cameron, of the firm of Cameron & Duncan, Dentists, Cincinnati. As a Mechanical Dentist he had found that Allen's plan did not stand the test.

Deposition of Aaron S. Talbert, Lexington, Ky., graduated in the Ohio College of Dental Surgery, in 1847. In 1852 purchased an Allen right. Does not believe Allen's plan good.

Dr. S. A. Main, residence No. 32 Bond St., New York city.—I have been a Dentist for 17 years. The only flux, a while since, in general use was glass; borax I never heard of for the purpose. (The mode and process of making blockwork, previous to 1851, on gold and silver plate was minutely described by the witness.) The subsequent use of platina for plates has made it necessary to have supports, either by arches in the shape, or by rimming and back plates. The first specimen of this new work came accidentally under my observation in 1846. A female stranger brought to my office, nine years ago, a set of teeth for repair—there had one tooth been broken off and the question she asked was, could I repair it. The work was novel to me, but I engaged to do it. I took my flux and pot enamel and succeeded in placing on the fractured part a new tooth. The general appearance of the specimen was, that single teeth, made out of solid body, were covered at their base inside and out with a gum body. It was not blockwork, but continuous gum work. I saw no other fastening to the plate but that of the gum body. I never saw the lady or the teeth afterwards. I have seen pieces in the mouth and in the possession of Dr. Dodge that were similar to that brought to my office by the lady spoken of above. Dr. Dodge told me that he had left the profession five years previous to our interview. The pieces I saw in his office were after he had left his profession. They were single teeth united by the gum and by that to the plate which was of platina. I knew of no other pieces in the mouth. I am not certain, in one instance, that they were not carved; but I know I have seen pieces made by Dr. Dodge that were single teeth. I have made continuous gum work; I have experimented for the last six or seven years, since I saw the specimen which I had repaired for the lady stranger. I made no practical work. I had, however, one under set, which I made prior to the 25th of July, 1850. I allowed it to be worn two years before I entered it on my book as "teeth in the mouth." It was on platina, single teeth united by continuous gum, had no back linings nor rivets, but the gum only fused on plate and around the teeth. The gum compound was of a body same as for single teeth or block work—of spar, silex, and kaolin—put in more glass and grind up in a wedgewood mortar, to make the pot enamel to flow easier. This enamel I bought, or it was given to me. The work thus worn two years in the mouth was taken out, not for any fault, but to put in a new set that set easier. Have read Allen's specifications; made an experiment on his formula, published in the Dental Recorder. I took the ingredients there cited—weighed them out—ground each separately, then altogether and wet up to paste. Being told, in the language of the specification to arrange the plate in the "usual way," I attached the teeth to a *gold* plate. To gently evaporate the water in the material it was gradually brought near the furnace. The little "joker" was put in and submitted to proper heat, and soon the material was displaced, the plate having melted away. I next hunted up some scraps of platina and tried once with that kind of a plate, but the compound when heated resembled parched corn when it has popped—it was all tied up in a knot. In 1852, a man named Stephen Wilson, in my office, pulled out his artificial teeth, part of a section united to a gold plate by backing, and a continuous gum, the owner said they were made by Beers & Morgan, Rochester, N. Y. They were in practical use.

*Cross Examined.*—(A publication of Dr. Main was shown, which he said was written by him.) I noticed the teeth brought by the lady, with some particularity, as they were novel to me, greatly different from any I had ever seen before. It was a whole arch, upper denture. She said, the work was made by Dr. Dodge, according to my recollection. I am not positive. I did not ask her name or residence. I talked with Dr. Dodge myself and saw several specimens else about the city.

Here Mr. Stanbery read a flaming card by Dr. Main, dated Dec. 1851, entitled in capitals. "*The Greatest Discovery of the Age.*" The witness passed through a very searching examination. Mr. Stanbery used his cross questions with much force, on the point that Dr. Main had in a sworn answer in chancery, affirmed that he had not made any continuous gum work on Allen's plan, which was practical work; Allen having sued him for non-payment of a note given for a right. I account for my statement that I had no practical work in use, while a gentleman was wearing a set I had made, by meaning by the phrase "practical work," work made to order for a particular person, and the specimen was taken out of the mouth before my oath was made. This work was but experimental work, it was put in the gentleman's mouth to see if it would answer—it was not made on the order of the patient. I was at the New York State Society meeting in 1851 or 2, Assur had worn a set of teeth which had given way, and as he wanted a new set, I determined to take a cast of his mouth and experiment on making his of the continuous gum work. At the time I took his impression, I had not previously made any practical work. I made one set on his old plate which he had left with me and a new set also.

Dr. Hayes, Buffalo.—Audibran says that porcelain teeth manufacture was learned from Reaumer's establishment of porcelain ware so famous at Sevres, France, in 1728. Duchateaux, an apothecary of St. Germain in 1776, the first in modern times, continued the work. Audibran, in 1821, received for his skill in their manufacture, an award from the Academy of the Fine Arts. The general mode of Dentists was to take feldspar, silex and kaolin to make the porcelain body. The enamel contained, in addition, glass, pumice stone. The ingredients, being ground and mixed, were moulded into shape of teeth. The enamel is composed of feldspar, a glass used for flux, and ground into a consistency of cream. Into this the teeth were dipt. They are placed on a slab of fire clay, into a fire clay, half cylinder, flat bottom muffle which is placed into a fire clay furnace. This is heated so as to vitrify the enamel and semi-vitrify the body. Block work undergoes the same process. The mode of setting dentures, prior to 1850, was by attachments to ordinary gold and silver plate. Borax is known as the borax of commerce in a crude state. Without backplates, platina plates won't answer a practical purpose. Porcelain is of two substances—one is fusible the other is nearly infusible. To work this, clay has to be put in, and kaolin is the purest kind of clay. As a flux, borax was not used until latterly. It is not many years since Dr. Wildman came to my office, he used borax. I don't know of a previous instance. He used it in the enamel and did not say if he used it in the body. I could not do the work by the patent specification alone. I would take part that would fuse at a high temperature, and another at a lower temperature. When following directions literally I would know I was not pursuing the object to be attained. My previous acquaintance in making glass of borax would have taught me that crude borax would swell or puff up. When I first read Allen's formula in Hunter's pamphlet, I thought it was garbled or not sufficient directions given to one skilled in the art. I wrote to Dr. Allen direct to send me some of his cement that I might test its quality. I waited a reasonable time for an answer and received none, when I proceeded to experiment myself. I took the recipe and as if life depended on my accuracy, proceeded to its test. I made a small mass, the compound was introduced into the muffle and furnace—it went in, the size of a hickory nut, and came out as big as a butter nut, and as porous as a honey comb. I then mixed a glass of borax, which fried out on the surface, and the mass was not baked at all; it had not chemically combined and I concluded that it would not suit for continuous gum work. I then tried the first mixture, with the crude borax first fritted and put in no gold or platina scraps. The mass was of the size of a hickory nut and in it was placed a molar porcelain tooth. If the mass would contract the same as the tooth, it would come out with no check or fissures. The result was, it shrank more than the tooth, it had from two to five checks. I found it necessary to make a frit altogether. I took glass, borax and enough spar so that when fused, acid would not work out the borax; then I took enough silex, kaolin, and fused them at a high heat, ground and mixed to a high heat. This was for a gum body.

When I first read Allen's specifications, I thought he had found something that would fuse on gold or silver plate or on most any plate. In following the directions merely, I should have used either gold or silver plate, for a flux might then have been attained which would have flowed on gold. By reading, we could not know but that the patentee had secured this. I should in justice to the words of the patentee, have to experiment as he directed, until I should find it to be a failure, which shrewdly enough I had guessed myself. As I had heard 20 years ago, that a man in Albany, N. Y., had succeeded in putting teeth on to gold plate in a heated furnace, I did not know but what he had secured this object.

I have read Dr. Hunter's work and experimented on his process. His compound is essentially different from the siliceous compound described in the Letters Patent; that makes a cement, while Dr. H's. makes a granulated body mixed in with cement which flows around and between the interstices, making an iufinitesimal series of arches.

When I showed Dr. A. samples made after his mode—Oh," says he, "I don't use that now; I have made an improvement and give *private* recipes. (Here Dr A., who was sitting near one of his Attorneys, Mr. Stanbery, with some emphasis of voice, and bringing his hand down on the table with violence, declared "he never had uttered such words.") Dr. A. showed me sets of pieces made by his revised recipe, which he did not communicate to me. He did not relate to me what his improvement was. Instead of plaster and sand or kaolin, I used asbestos once, and part of it cracked. I never met with an accident with plaster and sand. Fitch I read years ago. I experimented on the Delabarre formula; I prepared materials and proceeded as with Dr. A's.; it made a composition which did not crack around the tooth; this was done without fritting, but the body did not please me; I then fritted and realized a decent body. I did not prefer the process and would alter it as I did A's. in using Dr. H's. plan. If the results were like mine, better work can be made from Delabarre than from Allen. Fitch's is a poor and blind translation, bad English; Delabarre's directions in the original, are more explicit than those of Dr. Allen. Work without back plates I think would not be of practical utility. He was shown a specimen; thinks there would be necessary one plate to fit the mouth, and another to brace it.

*Cross Examined.*—I have read Fitch's translation of Delabarre in his Dental Surgery, and have heard Wildman's translation read in court. He cited the passages of the former which he said would sustain his assertion that it was bad English, but not that it did not convey the author's meaning; still it is clearer than the directions of Allen. I do not think in reading the latter, a skilful manipulator in block work would have known that gold was not intended. As to the borax, he would necessarily know that crystal borax should be used, and that by following the patent, the work would be useless and hence the direction was a mistake—the formula calling for one ounce of borax, (crude,) if he used fritted borax he would be in the dark as to the quantity intended. The revised instructions of Dr. A. differs from those of the patent; they direct the use of other ingredients, added in the siliceous compound. Dr. A. told me he did not continue to use the ingredients exactly laid down in his patent, but after a "*secret recipe*,"—(Dr. A. spoke out, "I *never* did.") I think that was the very expression. Hunter's and Allen's differed in their properties; Allen's cement would fuse alike, being a compound; Dr. H's. not, they having been fused separately first. I

think alumina melts; it separates into parts. Allen's and Hunter's would not come out melted alike. If the recipe of Allen was presented, and $1000 depended upon my attaining a successful result, instead of following literally its readings, I should follow an old recipe of my own which I had tested. I should have fritted the borax.

*Re-Examination.*—Common alum is the sulphate of alumina and potash ; alumina is soluble in water. If the life of a patient was at stake by following the formula, I should of course do it.

Dr. J. Doughty. When I gave my deposition in this case, I had not tried Dr. Hunter's formula ; have done so since in his laboratory. With Dr. A's. formula there is difficulty in fusing on to the plate ; it requires several heatings. I have made two specimens by Dr. H's., and they had no checks or flaws, and only required one heat for the cement and one heat for the gum ; one of the specimens were exhibited.

*Cross-Examination.*—My experiments after Dr. A's. plan was in 1852 ; this by Dr. H. was within four or five weeks. Of the former I made perhaps 20 specimens ; of the latter only two. Could make Dr. A's. work at the first experiments as well as at the last.

Wednesday Morning, May 16th.

Dr. Geo. A. Hays, Recalled. I wish to correct my statement made yesterday ; I should have attributed to Dr. A. the remark "private" not "secret" formula. The latter expression I take back.

The witness having by consent of the Defendant and the Plaintiff been selected on the evening previous to examine the artificial teeth of Dr. Taliaferro, made by Dr. Hunter, reported, that it is a specimen of continuous gum work united to the plate by a gum body.

*Cross-Examination.*—I now think the words used by Dr. A. were "private recipe ;" he said he gave it to those who had received or were receiving his patent. I did not learn what the private formula was. As to the set of Dr. Taliaferro, it's my opinion that the gum body was fused on, when the set were made. The teeth are fastened by back plates, but if the gum were taken away, the teeth would yet stand on the plate. I saw no indications that the gum body had been put on, on some subsequent occasion ; if that had been the case, a clumsy job would have been the consequence.

*Re-Examination.*—I should characterise Allen's as a glass, and Hunter's as a porcelain.

Dr. David McKitrick. My deposition has been taken in behalf of Dr. Allen. I had previously been engaged in the laboratory of Dr. Van Emmen, who was connected with Allen in putting up work on Dr. A's. plan. Subsequently, I have been in Dr. Hunter's laboratory and have seen his work put up. There is a decided difference in their work and a difference in their manipulating. Dr. Allen has his furnace heated before-hand ; Dr. Hunter's is not put in muffle at that heat, but when cold, and it is then heated up gradually. There is a difference in mixing the compounds; A's. is in a fine powder and wetted into paste. H's. is granulated and fine powder mixed in, paste put round the teeth. The former requires at least two heats, and the cracks are filled up before the enamel is put on, and the latter only one.

*Cross-Examined.*—I never had a difficulty with Dr. A; but had a difference with Dr. Van Emmon, as he failed to comply with a verbal agreement made with me; in Spring of 1854, I went with Dr. H.; my deposition gave a high character to the work of Dr. A. as far as my experience at the time enable me. The witness described the muffle and the furnace of each; both were united with Dr. A.; I never had two heatings with Dr. H.'s plan; the same was observable in the process of Dr. George W. Kendall, who did the principal work of Dr. H. It is something to have to fill up the cracks and re-insert the work into the furnace.

Dr. J. M. Brown.—I keep a Dental Furnishing Establishment on the N. W. Corner of Fourth and Walnut Sts., Cincinnati, Ohio; I commenced it in 1846; it was the only one of the kind then, and the only one at the present time in this city; the first time I obtained platina plates for sale was in 1848, on the suggestion of Dr. Hunter, who came to me and said, he was using such, which he had to send specially for to the East, and he might as well pay me a profit as to the Eastern seller; he said if I should bring it on he would take such a number of ounces, and if others did not buy the remainder, he would take it all; I purchased and brought out the platina, before which none had been kept on sale here; I sold single teeth to Dr. H. in fall or winter of 1850, to make specimens of artificial dentures for the World's Fair in London. He showed me, not long afterward, two or three specimens; these specimens shown me, resemble them; I am not a Dentist, but I specially remember this double cavity in the plate of one; I described to Dr. Allen, who soon afterwards came in, what Hunter had made; he asked what the work was; I said it was nice work; he asked if it was block-work; I said it was gum-work, flowed around the teeth; I specify the time from my recollection, that the occurrence took place before I went East in February, 1851.

*Cross-Examined.*—I ca'nt identify the specimen exactly, but this one in every way appears to be the same one H. showed me before he sent his specimens to the World's Fair; I have no recollection of Dr. A. having, when he called in, as alluded to above, shown me a specimen of the result of his experiments; but previous to his visit, I did see work in the mouth of a Mr. Hatch, who was clerk at the Gibson House; it had on it continuous gum; he brought the man into my store the year before; the color of that gum was different from this; the teeth appeared to be single; I could see the backing and rivets through the teeth; the gum was of a purple color; A. told me of having tried to make work of this kind, and think that after

Hunter had requested me to bring on platina, Dr. A. got some and brought me back a slip of the plate on which was flowed a body, but no teeth attached ; I scratched it with a knife, and it scaled off easily; some parts of it adhered better; it was not very good, as now considered; he told me at the time not to tell any one of his success, and I faithfullyc omplied with his request, and never told any one. In 1849 he purchased articles, and said he was experimenting. Hatch has gone off.

*Re-Examined.*—This specimen looks more like that I saw in Hatch's mouth ; the color on the surface is all I could see ; I could not tell the composition ; this bears a strong resemblance in its general appearance to the Hatch set—(To a juror)—I think Hatch's was on a platina plate ; it was either of that metal or silver ; if it was silver, I might not have been able to distinguish it from platina.

*Re-Cross-Examination.*—Hatch appeared to be delighted with the set A. had made for him ; he was an old Batchelor, and seemed to have concluded that he then looked quite nice.

Dr. S. L. Hamlen.—I know Dr. A. ; he showed me a specimen of what was in Hatch's mouth; it was on platina or silver plate ; I thought it was silver ; its appearance was like that of Levitt's enamel, only more purple, looked like this, only more darkly purple; I dont know what it is; it is all one simple enamel; the color is all through it.

*Cross-Examined.*—Five or six years ago I saw specimens by him of the compound adhering to the plate until forced off ; he then said he was experimenting to make something flow around the gums, but did not say anything about sticking the teeth to the plate; the compound alluded to did not stick tight ; he did not tell me that he had succeeded.

Dr. H. Crane, recalled.—Dr. Allen some five or six years ago, showed me specimens which he described as being similar to that in Hatch's mouth ; it was a purple enamel, designed to flow over the plate to prevent galvanic action. Levett's agent applied to me to sell his receipt ; he showed me samples, and, on calling again, he told me that Dr. A. had bought a right for $200 or $300, I do'nt recollect which; this was perhaps in 1849 ; after this conversation, Dr. A. showed me a similar specimen; this one resembles it; it was all an enamel body, put on to serve as a body, but it was nothing more than an enamel upon gold or brass plate.

*Cross-Examined.*—A gum body could not be made out of Levitt's enamel ; Hunter called his specimens, an improvement in setting single teeth and connecting them together by a gum body; I never saw the specifications which he used in describing his work, (Here Mr. Stanbery animadverted upon the non-presentation by the defence of the specifications alluded to—Mr. Matthews said, for himself, he had never seen or even heard of them.)

The witness was shown Dr. Hunter's publication (p. 66, of his, description of one of the specimens sent to the World's Fair; this said he, is block-work.

Mr. Stanberry said deprecatingly for the defence, "Oh! that onr enemies may write a book."

Mr. Matthews retorted, "Some of our enemies can't write a book."

George W. Kendall.—Am a Dentist; have been with Dr. Hunter for 8 years; I commenced with him on the 1st April, 1847; he was then experimenting to unite teeth by continuous gum to plate; on my first day of service, I was put to grinding materials for block work in which he was then working; he was then making a set of block-work in three pieces, which required considerable labor and much skill; he said he wanted to avoid that labor and make the block-work all in one piece; this was the subject of his constant experiment; ordinary block-work contracted one-eighth; Dr. H. continued to succeed until he made a full arch, which was afterwards mounted on to the plate by solder; he proceeded from point to point until he completed a set of gum-work, soldering being unnecessary. I was present when these teeth were made for exhibition at the World's Fair; the following was the process; the teeth were arranged on the plate—with materials to sustain the teeth—the body was then filled in; they were taken off plate and put into furnace; the teeth were thus united by continuous gum, and soldered on to a silver plate, on which it could not have been fused by heat; the other specimen was a platina plate; the gum body was flown in around the teeth which had been riveted on the plate by being placed on a tablet of plaster and cement, and gold soldered by being placed in the furnace; the gum color was then applied and fused by being submitted to a less degree of heat. The teeth of the other specimen were made by Dr. Hunter himself, and put on the plate in the same way, which does not differ from the continuous gum work since made by Dr. Hunter; all of them were made for the World's Fair exhibition and sent there; the double set was sent by themselves; the other one a month or six weeks afterwards; on the close of the Fair, one set was returned with a tooth broken off, it was the eye tooth or the adjoining one; by the flux, I put the tooth on in the broken place; no other alteration was made in the work or change of nature or character of the compound or gum; the other two were returned as they are; there was no great difference in the time of making the several specimens; one set was sent early, as exhibitors were urged to send early by the St. Lawrence, the U. S. Government Vessel appropriated to carry articles from this country; one was sent, the other one retained for somewhat more elaborate finish. Complaint, I know, was made by Dr. Hunter, through Messrs. Jones, White & McCurdy, of the delay of Edward Sanford & Co., the transporters; when he learned that only one set was on exhibition, Hunter wrote to Sanford an *energetic* letter; E. S. & Co., got a letter from London partner, at the close of the Fair, that the package had just been found. The description in the publication referred to by the other witness, referred to a specimen which Dr. H. admits, which is one where the teeth are soldered to the plate.

I am acquainted with Steemer's formula. (The formula was shown.) I have put up a piece of work by it—this is it—on platina; these three specimens are made after Fitch's translated formula of Mons. Delabarre; that of Dr. Hunter, and that of Dr. Allen; the materials in two of them were fritted, the other were not; (nice work.) I have made experiments as to the relative contractility of the composition of each formula, and have sections of an oblong shape, placed on this card, exhibiting the original bulk before baking by the mark on card behind each specimen—as they have contracted the specimens show for themselves. Hunter's contracts the least; the appearance of Dr. A.'s and Dr. H.'s are the same up to the point of fusion, but not when cooled; Dr. H.'s is rough and dark, owing to its being a granulated body; while Dr. A.'s has a glassy surface, is a glass enamel. I have examined A.'s formula recited in the patent; followed it in my experiments; I put the teeth on with wax; brought it up to pure gold fusion heat; the borax for flux was used without fritting, and the result was, the work was porous and full of bubbles; I then fritted it and submitted it to the same heat as Hunter's, to coin gold heat, and this is the result. I have made work after Dr. H.'s formula, and these are some specimens; I sift after grinding, to the same size as grains of gun-powder, an l prepare two bodies, the flux, silex, borax and potash; I used calcined borax; it is same as the borax of commerce; after it is baked, it becomes calcined borax; this is a small specimen of the gum body; there was no shrinkage in it, if there was there would be cracks; this is the universal fact in making H.'s compound.

*Cross-Examined.*—I have no business connection with Dr. Hunter; I left him on the 1st of April last; some of the specimens shown were made last year, and some two or three made last week. As to the specimen sent to the World's Fair by Dr. H., I believe the set was united together by spiral springs. The Delabarre specimen shown by me was made last year. Its composition is as follows:

| | | |
|---|---|---|
| Porcelain paste, | 140 | grs. |
| Calcined gypsum, | 20 | " |
| Silex, | 8 | " |
| Red lead, | 25 | " |

The porcelain paste I made of

| | | |
|---|---|---|
| Silex, | 3 | parts. |
| Feldspar. | 6 | " |
| Clay or Kaolin, | 1 | " |

I do'nt know how much alumina may have been in it; there was some in the clay and the spar; fifteen to twenty per cent. perhaps in each; Delabarre says "porcelain paste"—that paste varies; I presume what I used was not what Delabarre used; American porcelain paste differs no doubt from that used in France, but still it is porcelain paste. I made experiments on A.'s plan a year ago, and made small specimens last week; this is the best specimen made after his plan; I tried the experiment with two heats, and the teeth fell off; unless other persons have more skill or better luck in following the specifications, good work can't be done by it.

The object of the early efforts and experiments by Dr. H. was, as he said at the time, to make a non-contracting body for artificial teeth, to be used in mounting—to make blocks in one piece, to unite to each other in one body without contracting. Six or seven years ago he succeeded. In 1849 he had such work in the mouth. These specimens were sent to the World's Fair, to show that improvement; one of the objects was to show it as a non-contracting body. The specimens were, two on silver and one on platina plate. They did not differ except in the mounting, one was with springs. The most important one did not get there; it was the most important for it was in advance of any thing which had previously been done. This pamphlet, I admit I wrote at the time, and don't retract any thing of it now.

*Re-Examined.*—The body is the same in each specimen, the mounting different, the contraction immaterially different. One of them after being baked, was soldered to the plate: the other two were fastened to the plate by the gum.

*Re-Cross-Examined.*—Hunter did not scout at A's. patent when it was announced, no more than it was a clap-trap. He contended that no mere enamel could hold the teeth to the plate, and A's. was all fritted together. Hunter claimed that A. had got his enamel from Steemer. Hunter's is not a mere enamel; it is a peculiarly compounded body, what no mere chemist would make, but what a mechanical dentist would make. H's. formula is not the same as that on which W. Fair specimens were made. It is now what it was in May, 1851. The original was published here, his granulated body, and copied in 1852 into Eastern Dental publications.

Further *Examination* in chief.—The scouting referred to was Dr. H. contending that no work could stand unsupported by linings or back-straps. Dr. Hunter had made practical work after his own plan as early as May, 1851, which was worn by Horace Nye.

Dr. A. M. Leslie recalled.—Prior to the meeting in Louisville, in 1849, of the Mississippi Valley Dental Association, Dr. Hunter confided to me that he had developed the entire principle on which this whole matter rests—the non-contractibility of a compound which overcomes shrinking, to be used in making continuous gum work. The full arch made was that sent to the World's Fair. He afterwards more fully developed his mode. The processes described to me by Dr. H. I recapitulated to the Association at Louisville.

Dr. A. then claimed a cement that would unite single teeth to the plate without solder or back-plates.

*Cross-Examined.*—After the Fair, H. developed more fully his plan, a porcelain body, without material shrinking, making an arch without a break bears some analogy to block work; the platina base was covered with a gold plate. Hunter made work on platina plate previous to the meeting of the Dental Association in Louisville, in Sept., 1851, say in May, June or July. The difference between a baked body and an enamel is, that the latter flows easier than the former.

At ten minutes of three, P. M. on Wednesday afternoon the oral testimony closed.

A letter written by Dr. Allen to the Editor, was read from pages 199–200 of the July, 1851 No. of *The Dental Register of the West.*

A letter written by Dr. Hunter, page 278, and the proceedings of the American Society of Dental Surgeons, in Philadelphia, page 283, *New York Dental Recorder*, Sept. No., 1851, were read.

Dr. Hunter's article, page 63, Dec. No., 1851 *New York Dental Recorder* was read.

Dr. Hunter's new method was read from page 1, *Dental Recorder* for Sept., 1852.

Dr. Hunter's communication to the *N. Y. Dental Recorder*, March, 1852, p. 151, containing Steemer's recipe was read.

At four o'clock the Defence rested their case.

### Rebutting Testimony of Plaintiff.

Deposition of Dr. S. S. Fitch.—Physician 25 years, and for nine years Dentist in New York ; translated Delabarre, first edition in 1829—2d edition in 1835. As to the part of the work pages 471–3, the method I know was not handed down to practical use. The French are given much to abstraction: when I was in Paris I did not call on Delabarre, but Dr. Brewster told me his work was not in practical use. I have expended $1000 myself in experimenting unsuccessfully by that formula.

*Cross-Examined.*—I speak and translate well the French ; my experiments were a total failure. I had to approximate as nearly as possibe to Delabarre; if he had perfected it, it would have been a great improvement. Retired from practice in Sept. 1836.

Deposition of Dr. John A. McCurdy, of Phila., of the firm of Jones White & McCurdy, manufacturers of artificial teeth.

I saw Hunter's set of teeth at World's Fair ; can't describe them, it was three years ago. I wrote a description of them in the July 1852 No. of the Dental News Letter, page 263. They appeared to be gum teeth and united by a siliceous cement. The article I wrote myself and considered it a fair description.

*Cross-Examined.*—Has reason to believe that another specimen was sent by Dr. Hunter. There was no key to the case and I did not handle the specimen.

Otis Aldrich.—I have resided in Cincinnati since 1847 ; in the winter of that year, Dr. A. made teeth work for Mrs. Aldrich. It was what Dr. A. considered his improvement ; a partial upper set, as I understood it, on platina plate ; I know it was on some white metal ; it was worn for two or three years. His experiments for improvements have been since 1840 ; his favorite idea was, to fix the plate and work together by fusion. Another idea he had was to restore the contour of the face. Dr. A. afterwards made my wife another set, a whole arch, the same which she now wears.

*Cross-Examined.*—I am brother-in-law of Dr. Allen.

Deposition of Dr. Edward Taylor, (taken by the Defendant.

Have been a dentist about 18 years ; know parties ; was connected with Dr. A. as agent to sell rights under his patent ; sold only a few ; can't recollect whether used patent specifications or not. Perhaps Dentists could make from the formula of the patent successful work ; platina plates are necessary by A's. formula ; cement would generally not answer alone ; in fusing, two heats were required ; thinks that a Dentist might succeed following the patent.

Mrs. Aldrich.—Dr. A. put in my mouth, about 1847, a set of teeth which I wore about four years. I had two natural back teeth left, and irritation was caused by the set ; the back teeth were taken out and a full plate put in on platina plate ; the gum was the new improvement of his, as he stated, they were similar work to the set I have in my mouth, which are a new set ; they are on platina and answer a good purpose ; the first set about four or five years worn.

*Cross Examined.*—The first set were made in 1847. Here they are ; they have been thrown around carelessly and the gum is partly knocked off. The second set, (with back-plates and rivets) were made a short time before patent received. An alternative set was made 18 months ago ; after the natural teeth were extracted, the new arch was put in.

*Re-Examined.*—Some of the gum scaled off before I discarded them from use.

To the Court. The present set were put in in the fall of 1851 ; she took them from her mouth and they were examined by Court and jury (superior specimens.)

B. F. Penniman.—I knew of the early experimenting of Dr. A., as I was in his office in 1846 ; either he or his father produced work similar to this, and said it was an improvement ; would create a change or revolution. The matter was of interest to me, for I had had my front teeth all knocked out and temporary provision made for their loss by Dr. Hunter, of Baltimore, in 1825.

*Cross-Examined.*—The loss of my teeth was when I was about 11 years old, from falling across a gate. I came here in 1843 ; went to New York and returned in April, 1846, and in September of that year visited Dr. Allen. The piece I saw there was similar, like this shown me; it was continuous gum on a plate, but I don't know how they were put on, or whether they had back-plates.

Wm. L. Goodin.—I wear an upper set of Dr. A's. work made near three years ago, I believe on the 3d of July, 1852. He made a set before which did not answer the purpose; they were gum teeth.

*Cross-Examined.*—Eighteen months previous to obtaining the 3d July, '52 set, in the winter of '50—'51, A. made me set of single gum teeth, which I used about 12 months; one tooth got loose; there is a fracture in the gum body when the tooth was out; Dr. Van Emmon made the set now in my mouth.

Dr. Wickersham—Resident of this city and dentist for 10 years; knew A. during that time and of his experiments. In 1847, I was employed in his laboratory, to mount teeth on gold plate; then he had succeeded in fusing on plate. I attended the Spring course of lectures in the Dental College of Ohio, during the summer of that year and attended on Dr. Allen. He was using platina plate; saw his specimens; there was union of teeth to each other, and to the plate, by the appearance, without rimming or back-straps. He continued to experiment, to make his compound more perfect. The outward appearance resembled a specimen shown; it had a proper coloring and made a handsome job.

*Cross-Examined.*—I studied my profession with Dr. John S. Liggett, in this city, in 1840. In 1841 I practiced through the country; traveling around to Dayton and other places. In the next fall traveled through Kentucky. In 1842, again in the city. I had no particular place for an office; I did my work at my boarding house. My work with Dr. A., in 1847, was gold plate work. I can't tell how many pieces were made. Several were put in the mouth; don't know if there was half a dozen. All I did was on gold plate. Dr. Duval was there part of the time. The continuous gum work was made of feldspar; the same as the tooth body; apparently the same. I never prepared any of the materials; did not see any of it; don't know what its color was before it was put on. Don't know what kind of a furnace Dr. A. used; *don't know what a muffle is!*

*Re-Examined.*—I had nothing to do with the tooth work. My department was plate-work. The process was kept secret; few dentists knew of it.

*Re-Cross-Examined.*—A's had no back plate; nothing held it to plate but gum body; not like the specimen shown.

Dr. A. Curtis.—Have resided here, mostly, since 1841. Engaged as a teacher and practitioner of the Medical art. I have known Dr. A. since 1843. I can't say when I first knew of his experimenting to attain a continuous gum. In 1843 I wanted one front tooth inserted, and I talked with Dr. A. about it. He was then in the habit of putting teeth on gold plate. He made me a set of front teeth and I wore them for some time. I then had all my teeth drawn and a new set inserted. He told me how to put in a gum—not wax, but permanent; would not corrode. How early he told me this I cannot tell. The first set were made for me in 1849; this was merely temporary. Before that he had intimated that he had been experimenting. I now wear a set made by Dr. A's process. I had one before, in 1850. I don't recollect of seeing specimens before the first set I had. I was interested, and encouraged him to thus imitate nature. He said that he intended to fix teeth on plate as block work; to be firm, but to be united. Previous to 1843 he showed me specimens of dental work, at the Fair in the Mechanics' Institute. I occupied part of the house, and was of the Committee on Dentistry. I don't recollect the character of the dentistry; expect it was blockwork on gold plate; don't think it was continuous gum work. His intent was to perfect the work. I conversed with him, at different times, and he said he was gaining ground. I tested his success in 1853, by the work in my mouth. It is the greatest improvement on old things I have heard of or seen. It is firm; don't yield in biting; keeps out improper matter; is not disagreeable, keeps the cheeks of an old man in good order, and aids articulation. I remember Dr. Alexander, of Mississippi, and that in 1851 or 1852, Dr. A. put an artificial set of teeth in his mouth.

*Cross-Examined.*—I believe Bonsall and Hunter sent specimens to the Mechanics' Institute, in 1843, as well as Dr. A. The '49 set was only for a temporary purpose; was on silver plate, I wore it until the spring of 1850. Dr. A. showed me, in 1850, specimens of single teeth, but not of continuous gum.

*Re-Examined.*—The teeth in my mouth have back plates and are rimmed; they are united to plate by a continuous gum.

Geo. L. Weed.—Resided in this city nineteen years, eighteen of which I was intimate with Dr. A.—living, for a long time, next door but one to him. Four or five years ago, I can't tell precisely, he showed work that pleased me, and a first set was made for my wife; three years ago he made a full set. He told me he had been experimenting a long time, and I knew he worked late at night in his laboratory.

*Cross-Examined.*—She wore the temporary set six months. They were block teeth, not gum.

Deposition of William H. Boehringer.—I have followed the occupation of the manufacture of artificial teeth about twenty years. Knew Dr. A. to have been experimenting upon a new method of setting teeth by means of a continuous gum. Saw a specimen produced by him in 1843: it was a small case, with from four to six upper teeth. The gum appeared to have been flown upon the teeth and plate, thns uniting the teeth to each other and to the plate, similar to the method for which he now holds a patent.

No Cross-Examination.

Deposition of Dr. James Taylor.—Dentist since 1827. Editor of the Dental Register. Know something of the manufacture of porcelain teeth. Know A's method practically. There were two methods previous to that. I used lining straps; A's method would not do generally, without lining straps. Gold plates were used before A's plan; they would not suit for his mode. Two heats are required for his body and one for enamel. If water was not previously driven off of borax, the work in A's plan would crack. Allen does not always follow the language of his own specifications. Reference was made to an article in April No. Register, 1851; that still contains my views; also to the July No., p. 200; to the proceedings of the Mississippi Valley Dental Association, in the Oct. No.; to Dr. A's practical work, between 1st Jan. and 1st April, 1851; his exhibition of specimens in 1849. Read the No. of April 1852. Does not believe Dr. A. had practical work before that he showed at the Association. Can't say to what extent De Labarre's plan was used practically; believe it was not practiced on. A. and H's plans are similar in principle, and designed to produce similar results; as near alike as the principles of "pull and push" in the movement of a wheel-barrow.

Judge McLean remarked, "These must be *original* principles.

The Report of the Dental Association Committee, at Louisville, was presented and read.

Deposition of Hugh McCullum, Augusta, Ky., taken in Feb. 1854.—Am a Dentist; know Allen since 1841 or 1842. Was directed to him for Dental work. Two or three years afterwards he told me he was trying to make material for a continuous gum without backplates. Saw specimens not near so perfect as his present work.

William B. Ludlow, a Dentist since 1849.—Studied with Dr. Allen in 1847 and Dr. Allen was engaged in experimenting then for continuous gum.

*Cross Examined.*—I assisted in experimenting; knew nothing of the composition; the plates were gold, sometimes palladium. Allen's experiments were mostly on parts of sets. Don't remember of parties names for whom work was made. Don't know about Levetts enamel. May have said when I left Dr. Allen that he had not perfected his plan. Allen's present mode differs from the old one used by him. The Patent mode of Allen I have not used in my practice yet.

Deposition of Dr. Thomas Wood.—Am Physician since 1839; was Professor in Medical College. Know both parties to this suit; saw Allen demonstrate his mode. I was pleased with his work; saw him experiment before the Dental students, by fastening two teeth by gum alone, without back straps, to a plate. The teeth could not be pulled from the plate by the fingers; I after a severe effort broke off one tooth above the gum. The history of the transaction was signed by Professors and students and published in Dental Register, Feb. 1852, pp. 120–1.

Deposition of Elisha Baker, of New York.—Have been Dentist for 40 years; have seen Allen's mode and don't recollect of seeing any work on that plan before that of Dr. Allen was introduced.

Joseph M. Locke recalled.—Alumina will not melt by ordinary heat, electricity or the power of the blowpipe might do it, flux don't melt.

*Cross Examined.*—Alumina enters into the composition of glass and glass melts. Silicate of alumina is silex and alumina. In this glass alumina becomes a silicate.

Deposition of Dr. Augustus D. B. Coleman, Newark, New Jersey.—Have been a Dentist for 12 years; have known Allen's method about three years. Have used it eleven months and find it preferable to any other. Consider *platina* plate necessary and did not know of the mode until practiced *by* Dr. Allen.

*Cross Examined.*—I had a right under Allen. Have never been familiar with the specifications, but learned in Allen's Laboratory. I buy his material and make my work with bands.

Deposition of Dr. Van Emmon.—Have been Dental partner with Dr. Allen; think his plan a good improvement. Saw first specimens of his five or six years ago. A skilful Dentist can make the work by following the directions given in the Patent.

*Cross Examined.*—A muffle is a small fire proof clay tray opened at one end. Allen perfected his plan about a year before the Patent. The work is not so good without straps as with them. When borax is used it must be *previously* calcined. A Dentist acquainted with the manufacture of block teeth would not know what plate would stand heat necessary on Allen's plan until he experimented. Gold, platina and palladium may be used under Allen's Patent.

Deposition of Dr. Clark S. Putnam, Dentist of New York sends a specimen of work done after Allen's plan without instruction; he purchased a right and in a few experiments, with his knowledge of the materials, succeeded. The specimen sent was made in fall of 1852.

Affidavit of Dr. James Robinson.—I reside at No. 7 Gower, St. Bedford square, Middlesex county, England; am a Surgeon Dentist, and have been practising my profession for twenty three years and upwards and still am carrying on that business. I have seen specimen of Dr. John Allens improved mode of mounting teeth upon metallic plates with continuous gums, which mode of mounting teeth I had never seen or known of before.

Deposition of Dr. S. W. Stockton, of Philadelphia, manufacturer of teeth, &c. Know both parties. Prior to '51, gold and silver plates were used—gold particularly. There are various ways of making fluxes: lead, borax, glass, tin, nitre, soda &c. are fluxes, glass and borax are the fluxes in the compound read to me. It would be necessary to reduce the borax to a glass of borax; without previous knowledge would not know that the equivalent of 1 oz. of crude borax, should be obtained in glass borax.

Dr. W. Z. W. Chapman, of New York.—There are two classes of Dentists—those who purchase teeth and those who make their own. Know Allen's specification. They are addressed to those, I should think, who are in the habit of making their own materials. Have constructed a set on Allen's plan. Back straps should be put on before the gum material. In 1853 I made, after Allen's plan, a set for a lady on platina which have proved good work.

Deposition of Edward Barlow, New York.—Is Dentist; knows parties. Have made work under Allen's patent. If 100 cases were tried perhaps 10 might not prove good because operators do not know enough. I have plates in use two years which are doing good service.

*Cross Examined.*—I have added other ingredients to the preparation made by Dr. Allen. I first tried gold plate, it fused. Sets need back plates, they are not practical without them. I got private instructions with the Patent.

Deposition of Dr. Kingly, New York.—Similar testimony.

Deposition of Morris Levett.—Considers that his enamel and Allen's artificial gum compound are entirely distinct, the object to be attained by each to be entirely different; regards Allen's invention new and useful. Platina plates for setting them upon artificial teeth were used many years prior to Allen's Patent. I knew that borax was a necessary preparation as a flux and have no doubt it was generally known to practical dentists who manufacture their own teeth or block teeth, prior to the date of Allen's patent. I knew, as "known fluxes," borax, oxide of leads, green bottle glass, window and white flint glass, and potasa fusa for dental purposes in the manufacture of block teeth, &c. I have no interest direct or indirect, in the event of this suit. Borax of commerce is usually kept in lumps. Frit or glass borax have always to be made and are not to be had in the shops.

Schedule *of the Patent issued to* Morris Levett *assignee of Morris Levett & Henry Davis* 18*th*, *Sept.* 1847.

"To all whom it may concern. Be it known that we, Morris Levett & Henry Davis, of New York, have invented a new and improved method of concealing the fastenings of artificial teeth and the plates to which they are attached, and we do hereby declare the following to be a full, clear and exact description of the same.

The nature of our invention consists first, in providing a coating of Japan or other substance to represent the color of the gum or skin of the mouth, and with this we cover the exposed surfaces and parts of the setting of gold or other material for fastening the artificial teeth and holding them in place, by which the exposed parts are made to correspond in color to the skin of the mouth and gums, and thereby prevent the exposure of the settings and fastenings when the mouth is open. Secondly, in the preparation of a varnish or other material and applying the same to the settings of artificial teeth, so as to cause them to represent the color of the mouth or gum. We prepare a compound which may be called a varnish Japan or other name as follows. Two parts of equal quantity of gum shellac and linseed oil and by heat dissolve the gum in the oil, a small quantity of the spirits of turpentine being put in to aid the solution of the gum, when these are reduced to a soft state they may be ground up together with oxide of bismuth, Chineese Cinnabar and Cobalt, in sufficient quantities to give the required color which may be varied by the proportion of each of these last named articles used. The whole being well worked together with a stone and mullet or pallet knife, may be softened with spirits of turpentine at discretion. The material is ready for the plates in the above state, and is then to be applied to the plates with a soft brush or otherwise so as to completely coat the desired parts with the varnish. The articles are then submitted to heat until they are baked perfectly dry and the coating then adheres to the metal as a part of itself. Coat after coat may be thus put on, one on top of the other, until the desired color and thickness of coating shall be obtained to correspond with the color of the gums and the mouth. The settings being thus coated to represent the other parts of the mouth, the teeth appear as if natural. It would be almost impossible to discern the settings when thus coated and inserted in the mouth.

Deposition of Dr. Hazlitt, of New York.—Have been a Dentist since 1844. I know Dr. Allen's method; his specifications are addressed particularly to mechanical Dentists. I used platina plates in my profession ten years ago.

Deposition of Stephen B. Wilson, New York.—Beers & Morgan made a set of teeth for me in 1849. I witnessed the process of making them; when put in the mouth a month had not passed before the gum cracked, in a year some of the teeth were broken.

Deposition of James H. Holt, New York.—Am Dentist. Saw Wilson's set of teeth. See no similarity to it in those made by Allen. Its composition did not resist the action of the acids of the mouth. It was on a gold plate with lining. The gum was put on as in Allen's, but the color and substance was the same all through. Allen's I consider useful.

Deposition of George F. Schaeffer, New York city.—Have been Dentist for fourteen years. Saw Wilson's teeth, they were not on Allen's plan, but on different principle. I don't know of similar work to Allen's.

Mrs. Bartlett, of Kentucky, sworn.—I reside in Covington, Kentucky. Have known Dr. Allen since 1846 or 7. I was suffering much from tooth ache at that time and had Dr. Allen to extract ten of my teeth. I again called and Dr. Allen took out all but six; finally I had all taken out. I saw artificial sets of teeth then made by Dr. Allen; their color was that of the natural gums. In conversing upon the propriety of having a set in my mouth the only objection I urged then was that the set was too clumsy and would be so heavy that I could not wear it. The sets were called "suction plate." A set, with springs, was made for me which I wore about six months, when I caught cold and my gums swelled. They were left with Dr. Allen, who put in a temporary set, having left the teeth in a bowl of water they were thrown out of the window by mistake. Dr. Allen made me a new set; the gum flowed in. Three years ago I had another set made.

*Cross Examined.*—I moved to Independence, Kentucky, in '46 or '47. When the teeth were taken out, the new set was put in place, in at least ten days. It was a silver or platina not a gold plate, I gave $25 for the temporary set; it had no gums, this was worn about 5 months, when a new set with gums was made for me while I was living at Independence. The second set was, as Allen said, on platina, which he galvanized. That set I wore, up to about two or three years ago; they had gums behind and in front. In 1846–7 Dr. Allen was preparing what would give satisfaction; he sent on to the East to obtain something that would harden the gum. She showed the second set which had been thrown out of the window.

ANSWER IN CHANCERY, UNDER OATH BY DR. MAIN.—READ.

The Dr. having been sued for a sum due on contract of purchase of right under Dr. Allen's Patent, made answer, in chancery, that he had made no sets after Dr. Allen's plan "except for philosophical experiments for his own instruction."

Deposition of Dr. Charles C. Allen.—Reside in the city of New York. Am a Dentist, in practice for the last twenty years. I was the Editor of the "New York Dental Recorder," in the year 1852. There was an article published in the Recorder that year, May No. page 271–2 Vol. 6, signed by Charles Steemer, in the presence of C. Burckhardt and Louis Doer; the original copy or manuscript of which is destroyed. The following is a correct copy of the said article, according to the best of my belief and recollection:

"Cincinnati, March 11th, 1852.

In justice to Dr. Allen I hereby certify that I am not the inventor of his new method of setting teeth with continuous gums and that the first specimens I ever saw, after this style of work was at Dr. Allen's office and this was before I ever attempted any thing of the kind myself. And although Dr. Allen did at one time try a compound I made for him, I am satisfied that he does not use it in his practice. I would further state that the injustice which has been done Dr. Allen through the public press by misrepresentations, was by the ill advice of others and I regret exceedingly the injury he has sustained in consequence of my wrong steps in the matter and I take this method of rendering to Dr. Allen justice and acknowledgement which is his due. CHARLES STEEMER.

CHRISTIAN BURCKHARDT sworn. I was in Germany, Treasurer of the Crown in Baden. The article in the Dental Recorder signed by me is correct. It is a true copy of one in German which I read to Steemer; it was his denial of a malicious card in the Gazette which he had caused to be published therein against Dr. A. Dr. A. did not employ me to secure a denial from Steemer. The correction was made in my son's store. I translated to him the article in the Gazette, whereupon he expressed great astonishment, that it made him say he was the inventor when he was not. He said he got his first idea of uniting teeth from the experiments of Dr. A. His suggestion and instruction no doubt, were of service to the experimenter and he had advantaged himself by Dr. A's. knowledge. Steemer had a patent for his enamel, but A. declared he could not make practical use of the Steemer enamel.

Deposition of CHAS. F. MUENTER, of Bolivar, Tenn. I was in the employ of Dr. Allen in his laboratory in Cincinnati, from the 16th or 17th January, '51, until the end of March 1852. About the middle of May I secured one Charles Steemer also to be employed by Dr. A. I had become acquainted with him but a few weeks previous. He told me he had been a miner in the Hartz mountains, where they have all sorts of ore, He here was out of employ and had been doing light work, driving nails into oil barrels at Burckhardt's Oil Factory, and had for awhile done enameling of pots in some foundry. I introduced him to A. in '51, not far from Seleg-mann's cigar store, on Walnut street, between 4th and 5th, in Cincinnati. I did this to procure a situation for a countryman out of employ, and that Dr. A. might obtain a workingman not quite as green as many others. Dr. A. employed him on account of his knowledge of enameling. Experiments went on by Steemer, A. and myself, of all the preparations that we could read of or knew any thing of. Steemer prepared a composition, but Dr. A. made no practical work from it. Dr. A. found out one of his own, better. It would be ridiculous to say that Dr. A. bought Steemer's formula.

*Cross Examination.*—The work that Dr. A. successfully made, I well recollect one particularly was on palladium,—but the principal plates used was platina. A silicious compound was used for the mineral gum; I do not recollect the formula. The metal union was gold solder, and afterwards fine gold; the union was by backings or linings in some; in others, they were strips—small strips attached to the last pin, and then covered over on the inside to make the inside smooth and natural. The strips were soldered to the back plates. This work was made, as far as I recollect, for Mrs. Dr. Irons, and also for a German grocery keeper, who lived up near the intersection of Front & Columbia sts. A few, I believe, were made on plates of finer gold than the ordinary plate, which is generally 18 carats fine. The alloy was platina; ordinary alloy used is not sufficiently stiff for this kind of work. Steemer was, perhaps, not longer than two months in A's. employ.

*An article in the Cincinnati Gazette,* dated Oct. 17th, 1851—alluded to above, was read.

Deposition of Emory G. Darling, read. The article in Dental Recorder, alluded to by Steemer, is correct. Have not put up work by Steemer's plan, but have done so by Allen's. Steemer's is worthless, however, and Allen's good.

*Cross-Examined.*—Have witnessed Dr. A's. work in the process of production.

Deposition of James T. Irwin. Am a dentist, and was employed with Messrs. J. & J. Taylor. Have made work on Allen's plan; never saw any made without back-straps; I was present when Dr. A's. specimen was tested in the Dental College. Dr. T. Wood got up the article which gave the account of the proceedings. Delabarre's formula has been discarded by the profession.

Dr. Miller recalled. As to the impalpable powder compound of Dr. A. and the granulated body of Dr. H., I make neither; I use a formula of my own, making a compound of medium fineness. The coarse preparation is better for block work; the finer adheres better to the plate. I prefer the finer; it shrinks less. I have put up between two and three hundred pieces of continuous gum work, and of these less than a dozen after A's. plan. I prefer the granulated body with only one heat in the furnace, to the fine preparation that requires two beats; the granulated body does not come down with perfect adhesion to the plate.

*Cross Examined.*—I never made more than half dozen specimens after my plan, which has not yet been disclosed to the public; have not made more than a half dozen after any one exact plan.

The publication of Dr. M., in which he speaks laudatory of his own new mode, was shown. I have made good work which has stood well by this plan of mine, but I have virtually abandoned working after the formula. This is similar work to that put up by mine.

*Re-Examined.*—It is substantially after Dr. A's. plan. Dr. A's. is the original; as a standing point I started from him—to him I am indebted for the attainment of what I call my own improvements.

Deposition of Dr. Samuel Wardle. Am a manufacturer of artificial teeth; am acquainted with Dr. A. and with his patent work. Have followed his specifications without instructiens, and made his works, (specimens here exhibited.) This was made without instructions ; I did not use scraps, but soldered platina back-plates, previous to the application of the gum body. I made the gum color by a recipe of my own : I first formed a glass of borax out of crude borax. As a dentist, heretofore I had not used platina, but had used palladium ; I think that a person used to the manufacture of block work could frem the specifications readily make work on A's. plan. Back-plates would make the work stronger ; a competent block-workman would know platina should be used. A person following strictly the directions of the patent *could not succeed if he used gold plate.*

The depositions of Messrs. PEEBLES, KENDALL, MCKITRICK, DUNCAN and WHEELER, were offered, but were not read, and hence we give no abstract of their contents.

On Friday morning at 10 o'clock, Dr. A's. attorneys rested the case.

Judge E. NEWTON made the opening address to the jury, for the Plaintiff:

It is necessary, gentlemen of the jury, to remove the irrelevant trush before us, which has rather embarrassed than benefitted your decision in this case. Here is a pamphlet got up and thrown broadcast in this community by Dr. Hunter, of an inflammatory character, which inflames the public mind and denounces Dr. Allen, branding him and charging him as fraudulent pretender, and perjured claimant, an ass, an ignoramus, wanting in scientific knowledge, in experience, in the integrity a necessary quality to invent—I speak advisedly, and the document itself bears me out in saying, that the palpable purpose of Dr. H. was to prejudice the claim of Dr. A. in this suit ; perhaps you have some of you seen this publication. I have no personal feelings in this matter, have a friendship for Dr. H., but professional duty requires you and I who alike are under oath, to be honest to ourselves and to the parties at issue.

It is your duty to discard feeling in this matter—friendship is commendable—here it has no control ; truth is your object and should be your guide in the jury box. If you have read this, the most singular of all documents, discard the remembrance of it from your minds. I know the public mind has been poisoned ; that publication contains the most extraordinary sentiments ever uttered by a living man.

(A juryman rose and protested that he had never seen the document and declared that he never had heard of the case before.)

As to a personal controversy between these two men, Dr. A. has had no controversy; only in one or two instances did he feel it his duty to reply. On H's. side it has been all abuse. After this suit was commenced, this defendant in the publication alluded to, made use of the most offensive remarks—"I am ready to meet you, &c." —"tool of a faction,"—"more tribute money,"—"and that too by false pretences,"—"patent monger,"—"I will give my head to the profession for a football if I fail to invalidate the patent,"—"war to the knife."

This same man has not one tithe of interest in the success of this suit, which he denounces was fraudulently brought into Court. Yet he has persistingly labored to break down Dr. A. to defeat the well meant intention of the inventor of a valuable aid to human happiness. Dr. Allen's plan is designed to reorganise, to build up anew the human organ; he has drawn from the earth whence man was originally taken, the materials and has so combined them as to reorganise the furniture of the mouth, one of the most important to our health and usefulness.

The declarations of parties to this suit—the pamphlets written, enter into the formation of the general character which peculiarly distinguishes this case. Its issue is of great importance to Dr. A., for he stands branded in those pamphlets as guilty of stupendous frauds. He is charged with having obtained the patent itself by fraud. The all of Allen depends upon this issue—he has spent 20 years of his life in perfecting an invention which he here to-day claims protection from designed and determined infringement.

Dr. H. declares that "he will defend the case out of principle"—there is but little practical reason wby he should be so interested, for we claim none but nominal damages for the purpose of establishing the patent. An important element of the suit is, that the people themselves are directly interested in the maintenance of the patent.

Ours is a patent for a new mode of setting mineral teeth by a fusible silicious cement. You can set the teeth, but the substantial cement must not be used—our patent protects us from others using any other combination of our cited material that will produce the same result. Our specifications are delineated in a palpable manner.

Perhaps you may think there is a difference in the quality of the several specimens of the work on both sides presented to you.

This patent was granted on the 21st of December, 1851. The suit is for tort, in pirating our right—the plea is not guilty. The defence give a long special answer, containing many points—one is, that our specifications are indefinite and were so intended to mislead and defraud the public. As to the "known fluxes," those that are applicable we have abundantly proved; as to throwing off the water of crystalization from crude borax, the testimony is overwhelming.

Out of a large number of depositions (naming them) taken for the defence, where the deponent was cross-examined by us, they have read but a single one of them; they did not dare to read them; do'nt let them talk about specifications made to defraud, when they suppress documents that would militate against their case.

They make much of the necessity of their being "back plates." Our patent specially provides that "they may be used." There is no fraud in that specification, for that contingency was provided for; back plates makes the work stronger, and is decidedly preferable; but the great magnificent idea was the one arch attached to the plate. The successful result of the great idea he will sustain in this patent, if he is not broken down by such trash as Leslie and the kindred conspiracy.

This great idea, carried into successful realization, has pervaded all lands, it is the wonder of Paris and London; here is a gentleman from London, testifying to its supreme excellence, and we have read the deposition of a celebrated London Dentist, as to its superiority.

Back straps make work more strong; but see Mr. Goodin, that strong, stout man, over 200 pounds in weight, whose teeth have stood for years. Experience and practice has condemned the objections and confounded the falsehood of the accusations.

This great scientific invention is worthy of support. Science has her trophies in all the practical walks of life. The most useful invention is often entangled with rude opposition. The inimitable discovery and valuable application of Morse, in the Electric Telegraph, was with pertinacity fought against, but the right was sustained.

Dr. A.'s oath in this matter is to be regarded, that is not to be consider a nullity. His reputation here, his whole life confute the bold assertion that he made a fraudulent claim of being the original inventor.

That smooth-tongued witness linked with H., said that Allen told him he had a *secret* formula—that man was nervous when he saw Allen's immediate and emphatic negative, and he did not leave the court-house until he recanted—that man's conscience smote him, and next morning he returned to the stand and made a correction of his words.

Fraud has been charged against A., but not an iota of proof of it has been presented.

This discovery has been made in your city—keep that prominently before your minds—either by Dr. A. or by Dr. H. In seven depositions taken by H. himself, the credit is given to A.—all say A. is the oldest experimenter—he had experimented night and day from year to year, while H. was a mere boy, a child. Who gives H. the credit of the invention? Among the whole of the assembled Dentists of the West, who knew both A. and H., who of them all spoke in favor of H. but Hunter's pet, Dr. Leslie, one of the bitter enemies of Dr. A., and an avowed advocate of breaking down all patents—a very tool of Dr. Hunter.

All here where both were known gave A. the credit—the Dental College, all the old Surgical Dentists. But H. determined to be the champion before the public. Off he skulked to Washington City, and obtained a copy of Allen's caveat. He shrank from going there to claim a patent for himself, but assumed to be the Goliah of the anti-patent party. He essayed to write A. down, and destroy his character. Hunter caught at a shadow, at a trifle, and lit upon the witness George E. Haws, he got from him a garbled statement as to Wilson's set of teeth; but we having closely cross-examined him, the deposition was suppressed—an instance of fraud itself.

There is Hays, the champion and bosom friend of Hunter—he it is who finds fault with the English of Fitch, the translator, a scholar and traveller. Then there is the specimen of Dr. Lawrence Bristol, of Buffalo, N. Y.; its base is mud, and the whole mass is mud; (this specimen was not on plate but on a Plaster of Paris model.) Bristol, Harrison and Hays, a beautiful trio!

There is George W. Kendall—this is a mighty strange man—those were strange doctrines for an intelligent man, promulgated by him in reference to the faculty and students of the Dental Class—that was an infamous paper. This is the kind of man that comes in to defeat the most valuable patent in the United States. That K. comes in here and presents specimens of pretended work after A., H. and Delabarre. I never saw such consumate fraud in my life.

Where is John T. King? He does not come into Court. He is among the "steemers."

There was Main; you saw him; he was one of those kind of peacock men—tail longer than the head. Stnff such a man with a good dinner and he will do what you want he should; and there is no greater a stuffer than this man Hunter. If that man was indicted for perjury he would not escape. His words are not true. In his great card he speaks of his own invention "as the greatest invention ever made." The man who makes false statements as to one thing is not worthy of confidence as to others. If false in one he is likely to be false in all.

Then as to Dr. "Laing"—his statement is a falsehood. He did not know that it had been used by Dr. Dodge.

As to priority of the invention Dr. A. is ahead of Dr. H. five or six years. Allen, as Aldrich said, has been experimenting since 1839 or '40. He made specimens four or five years earlier than did Dr. Hunter. He don't go behind 1846. His work was not in the mouth until 1848; his communication to the public says in 1851. He is behind A.; for the latter made work for Mrs. Aldrich, his sister in 1847, and for a lady in Covington, in 1846. Allen has the priority in conception and also in putting them to practical use. But he who first conceives the idea is entitled to the invention; even if subsequently another applies it before he does. But this case does not turn on that point; that is not the case here; (See 1 *Story*, p. 600). This puts an end to all the difficulties in this case; if in the race of diligence H. got first into practical use, A. being the first in conception, must be protected in the patent that he has secured.

As to the infringement, we must prove that he has infringed. I

had supposed that they would not attempt to controvert our position; the infringement occurs when he makes due preparation himself or causes it to be done by others.

Dr. H. in his own publication admits that he has infringed, and prides in it. He says that he and the whole profession may use the formula and method as they please. H. brings in here the very specimens he has made. Mrs. Stephens says her work was made after the patent. Dentists unequivocally say that his work is the same as ours. It is substantially, for it attains the same object. (See decision, 15 Howard—where five out of nine Judges, of which Judge McLean was one, gave their decision.) It is so laid down as law by Curtis, if parties have the same object in view, the change of method, as that of a granulated body, does not relieve the party from liability, for infringement—if he betters it—makes an improvement, which he does not patent, and in this case we do not admit H. has made any improvement; still the patent must protect its owner.

Let all the specimens, those of A. and H. be taken to the jury room and be mingled together, you could not distinguish them apart. The witnesses are numerous as to infringement.

As to its usefulness, there is an overpowering array of witnesses; the case is established, so far as that point is concerned.

Mr. J. M. Locke analyzed all the articles by scientific rules, by the laws of nature applicable to matter—as to the points where the two harmonize—that law found out, is as unerring, universal and uniform as the revolutions of the planets. H. has different proportions of the materials, but the same result is secured, the same ends effected. There is 25 per cent. more kaolin in Delabarre's than A.'s. ; 1,500 per cent. more heat is required. Locke says it is impossible to make effective work from the formula of Delabarre. We introduced Fitch himself, and he says it is impossible to make work after Delabarre. Putnam says the same. As to the Burr specimen.—One person says that another person told another person that the curious set of teeth presented were those of Burr. Dr. Jobson says he can't tell you if it was ever used ; in Paris, he says there was none of the Delabarre work—persons in the profession there had got tired of it.—As to Steemer, now gentlemen, that matter is really ridiculous ; but as it has been made so prominent—that whole misrepresentation, base conspiracy, has been exposed in a recantation by Steemer, in the columns of the Cincinnati Gazette; when the first article appeared as an advertisement, and as Steemer himself said, written by a medical gentleman.

This conspiracy with Steemer, has been playing the game to defraud and destroy Allen. They at first palmed Steemer off as the original inventor, and tried to take all the credit from Allen. At one time Steemer himself claimed to be the inventor, and then he became a conclusive witness against himself.

